# Structural, Ionic, and Electronic Properties of Solid-State Phthalimide-Containing Polymers for All-Organic Batteries

**DOI:** 10.1021/jacsau.4c00276

**Published:** 2024-06-07

**Authors:** Riccardo Alessandri, Cheng-Han Li, Sheila Keating, Khirabdhi T. Mohanty, Aaron Peng, Jodie L. Lutkenhaus, Stuart J. Rowan, Daniel P. Tabor, Juan J. de Pablo

**Affiliations:** † Pritzker School of Molecular Engineering, University of Chicago, Chicago, Illinois 60637, United States; ‡ Department of Chemistry, Texas A&M University, College Station, Texas 77842, United States; § Department of Chemistry, University of Chicago, Chicago, Illinois 60637, United States; ∥ Artie McFerrin Department of Chemical Engineering and Department of Materials Science & Engineering, Texas A&M University, College Station, Texas 77843, United States; ⊥ Artie McFerrin Department of Chemical Engineering, Texas A&M University, College Station, Texas 77843, United States

**Keywords:** redox-active polymers, multiscale modeling, all-organic batteries, molecular dynamics, organic mixed conductors

## Abstract

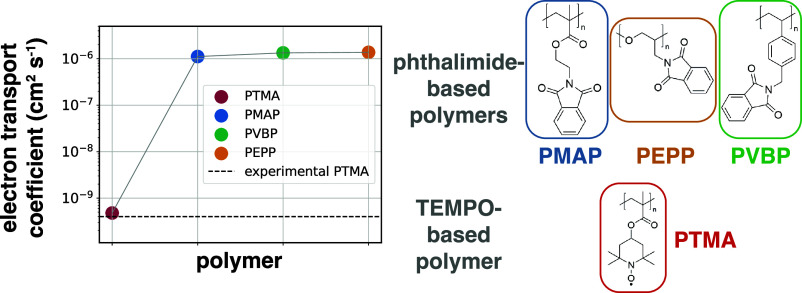

Redox-active polymers serving as the active materials in solid-state electrodes offer a promising path toward realizing all-organic batteries. While both cathodic and anodic redox-active polymers are needed, the diversity of the available anodic materials is limited. Here, we predict solid-state structural, ionic, and electronic properties of anodic, phthalimide-containing polymers using a multiscale approach that combines atomistic molecular dynamics, electronic structure calculations, and machine learning surrogate models. Importantly, by combining information from each of these scales, we are able to bridge the gap between bottom-up molecular characteristics and macroscopic properties such as apparent diffusion coefficients of electron transport (*D*_app_). We investigate the impact of different polymer backbones and of two critical factors during battery operation: state of charge and polymer swelling. Our findings reveal that the state of charge significantly influences solid-state packing and the thermophysical properties of the polymers, which, in turn, affect ionic and electronic transport. A combination of molecular-level properties (such as the reorganization energy) and condensed-phase properties (such as effective electron hopping distances) determine the predicted ranking of electron transport capabilities of the polymers. We predict *D*_app_ for the phthalimide-based polymers and for a reference nitroxide radical-based polymer, finding a 3 orders of magnitude increase in *D*_app_ (≈10^–6^ cm^2^ s^–1^) with respect to the reference. This study underscores the promise of phthalimide-containing polymers as highly capable redox-active polymers for anodic materials in all-organic batteries, due to their exceptional predicted electron transport capabilities.

## 1 Introduction

Nonconjugated, redox-active polymers (RAPs) have emerged as a new class of electroactive materials.^1−3^ Due to their redox, magnetic, and charge conduction properties, RAPs can be applied for a broad range of applications from optoelectronics and spintronics to memory or energy storage.^1−6^ Particularly interesting is the use of RAPs as alternatives to metal-based materials for batteries.^1,3,7−9^ In this context, RAPs may offer more flexibility for chemical degradation strategies that would enable the realization of “circular” batteries.^10,11^ Moreover, all-organic batteries would offer a pathway away from the reliance of current battery technologies on metals such as lithium, nickel, and cobalt that present economic, ethical, and environmental challenges.^11,12^ A key hindrance in the development of RAP-based, solid-state, all-organic batteries is that the vast majority of solid-state applications using RAPs reported to date have relied on preferentially oxidized (i.e., p-type or high-potential) radical groups.^13,14^ However, preferentially reduced (i.e., n-type or low-potential) RAPs are required as active materials for the anode. Hence, developing n-type RAPs is crucial for realizing the promise of RAPs in applications such as all-organic batteries.

RAPs are, in principle, highly modular materials, and at the most fundamental level, their redox-active group governs their electronic properties while their backbone shapes their thermophysical properties. While numerous n-type redox-active groups have been used as molecules or dissolved oligomers in redox flow batteries, the diversity of n-type molecules appended to polymers and used in RAP-based batteries is primarily limited to viologen, quinone, or diimide species.^2,9,12,15,16^ Regarding backbones, a key finding has been that flexible macromolecular backbones with near-room temperature glass-transition temperature (*T*_g_) may promote electrical conductivity by allowing for thermal annealing treatments that lead to the formation of electronically percolating networks of redox-active sites.^17^ This was the case for the p-type, TEMPO-based (2,2,6,6-tetramethyl-1-piperidinyloxy-based) polymer PTEO.^17^ Due to its poly(ethylene oxide) backbone, it achieved a record-high electrical conductivity of 0.2 S/cm over length scales of <600 nm.^17^ A similar strategy applied to the galvinoxyl n-type redox group and employing a low-*T*_g_ polysiloxane backbone also favored electrical conductivity, although the bulkiness of the galvinoxyl groups likely limited the achievable conductivity to a still relatively high value of 10^–2^ S/cm.^14^ Overall, understanding how the backbone-redox group design at the molecular scale translates to the polymeric material properties remains challenging.

The use of RAPs as active materials for battery electrodes, as well as for any other application involving polymer interaction with an electrolyte (such as electrochromics and sensors), is enabled by their mixed ionic-electronic conduction properties.^18,19^ During charging, when an active material in the p-type positive electrode (n-type negative electrode) is oxidized (reduced), the redox active unit transfers (receives) an electron from the current collector, resulting in a positively (negatively) charged species. Simultaneously, anions (cations) “dope” the polymer at the p-type positive electrode (n-type negative electrode) to maintain charge neutrality. Upon discharge, the polymer is dedoped, and the neutral redox-active unit is restored. Hence, the polymer state of charge changes during battery operation, while the polymer also “swells” due to the uptake of ions (that most often takes place together with some amount of solvent^20^). Meanwhile, electron transfer within the RAP occurs by an electron-hopping mechanism, whereby electrons propagate homogeneously by self-exchange.^1,21,22^ It remains poorly understood what the impact of the polymer state of charge and polymer swelling are on electronic and ionic properties of RAPs, and how the dry-polymer backbone-redox group designs can be translated to electrolyte-rich environments.

While molecular modeling may help in our understanding of RAPs, studies have been limited, in particular in the case of n-type RAPs. Beyond TEMPO-based systems,^17,23−25^ a generic coarse-grained model has been developed to study charge transport in RAP solutions.^26^ To be predictive and distinguish between the performance of various polymers, these models necessitate detailed molecular-level information regarding the particular redox-active group as their input. Another type of effort has concentrated on molecule-specific characteristics by exploring various sets of redox-active units^27−29^ or oligomers^30^ using electronic structure methods in the gas phase. A limitation of these studies is that they do not account for condensed-phase conditions, which could potentially alter the resulting electronic properties. In summary, there is a need for chemistry-specific computational studies that address the complex interplay between the solid-state organization of RAPs and their ionic and electronic properties.

Here, we model solid-state structural, ionic, and electronic properties of n-type, phthalimide-containing RAPs using a combination of atomistic molecular dynamics, electronic structure calculations, and machine learning surrogate models. We choose *N*-methyl-phthalimide, or simply phthalimide, as the redox-active group as it emerged from a recent virtual screening campaign performed by some of us as a promising group to reach both the low redox potential needed for an n-type material and the high electronic couplings needed for high electronic conductivity.^28^ We attach phthalimide to several polymer backbones and probe how these modulate the polymer solid-state properties. We also determine the impact of two critical system parameters during battery operation, namely, the polymer state of charge and the swelling of the polymer in response to the uptake of electrolyte solution. We find that the polymer state of charge has a large impact on all properties and in a somewhat universal way across the different polymers, lowering the systems’ *T*_g_ and modulating phthalimide–phthalimide configurations and thereby impacting ionic and electronic transport. Differences though emerge between the different polymers, wherein molecular-level properties (electronic couplings, reorganization energy) and condensed-phase properties (density of radical sites, effective electron hopping distances) interplay to determine the electron transport properties of the polymers. While the poly(ethylene oxide)-based polymer shows the largest predicted electron transport capabilities by achieving a balance between electronic coupling strength, reorganization energy, and effective distance for electron transfer, the investigated phthalimide-containing polymers are overall very promising, possessing very high predicted diffusion coefficients of electron transfer on the order of 10^–6^ cm^2^ s^–1^.

## 2 Results and Discussion

### 2.1 Polymer–Electrolyte–Solvent Systems

**Figure 1:**
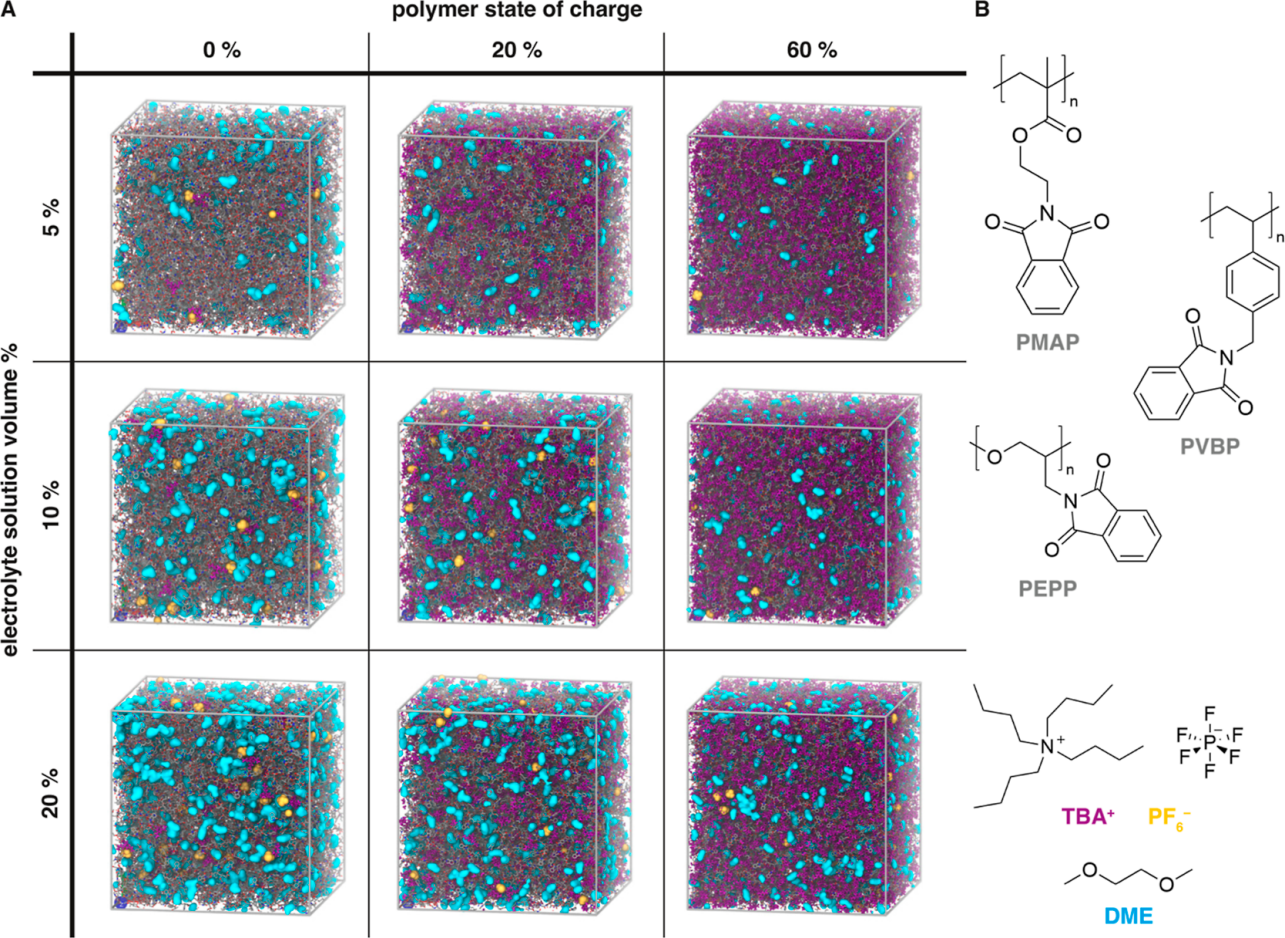
Systems modeled: phthalimide-based polymers at different swelling conditions and states of charge. (A) Renderings of the PMAP systems for the different swelling conditions (expressed as the electrolyte solution volume %; 5, 10, and 20%) and polymer state of charge (0, 20, and 60%) investigated. (B) Chemical structures of the components: the polymers [PMAP, PEPP, and PVBP; in gray in the renderings in panel (A)], TBA^+^ (purple), PF_6_^–^ (yellow), and DME (cyan).

The phthalimide-based polymers of interest for this work, with different backbones, and in different states of charge and swelling conditions, are schematically shown in Figure 1. We choose to investigate different backbones because their different segmental dynamics and size may lead to different structural (e.g., phthalimide packing), ionic (e.g., ion transport), and electronic (e.g., charge percolative networks) properties of the resulting phthalimide-containing polymers. In particular, we compare poly(methyl methacrylate)-, poly(ethylene oxide)-, and polystyrene-based polymers—poly(*N*-(methacryloxyethyl) phthalimide) (PMAP), poly(2,3-epoxypropylphthalimide) (PEPP), and poly(*N*-(vinylbenzyl) phthalimide) (PVBP), respectively—as shown in Figure 1B. Each periodic system contains 100 chains with a degree of polymerization of 30, corresponding to molecular weights of ca. 7.8, 6.1, and 7.9 kDa for PMAP, PEPP, and PVBP, respectively.

We investigate the impact of two critical system parameters—(1) state of charge and (2) swelling %—by varying them in a discrete fashion as shown in Figure 1A. During battery operation, a polymer constituting a solid-state electrode changes its state of charge or redox state. In the specific case of phthalimide-based systems, the low redox potential of this unit means that the material will be reduced. We model the neutral systems (i.e., state of charge = 0%) by having all the phthalimide-based monomers being neutral. We model the charged systems as follows: each of the 100 chains has the same amount of charge (e.g., in the 20%-charged systems, 6 monomers out of the 30 have a charge of −1, leading to a charge of −6 per chain), with charged and uncharged monomers randomly distributed for each chain (see Methods for details). The resulting net negative charge of the charged polymers is compensated by the inclusion of TBA^+^ cations. We simulate a range of swellings that is typical of experimental settings, namely, 5, 10, and 20% (expressed as the electrolyte solution volume %).^31^ We model swelling by including a volume % of electrolyte solution, namely a solution of 1,2-dimethoxyethane (DME) with 0.5 M of the organic salt TBAPF_6_ (tetrabutylammonium hexafluorophosphate), that is equal to 5, 10, or 20% of the total simulation box volume. For more details on this procedure, and the detailed molecular composition of all systems, see Table S1 and associated discussion. Once set up, all systems are equilibrated in their melt state (at 900 K) and then cooled down to various temperatures for analysis (see Methods for details). A schematic of the overall simulation protocol is given in Figure S4.

### 2.2 Glass-Transition Temperatures

First, we probe the effect of swelling and state of charge on the *T*_g_ of the polymers. We note that while the absolute value of the simulated *T*_g_ is higher than the corresponding experimental values due to the fast cooling rates used in the simulations, the atomistic force field recapitulates the ranking of *T*_g_ values observed experimentally for the bare-backbone polymers PEO < PS < PMMA (Figure S2).

**Figure 2:**
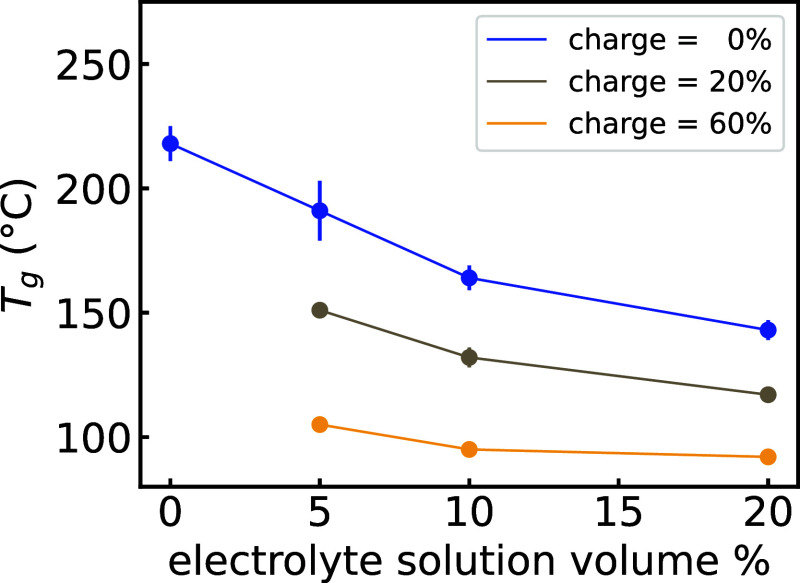
Computed *T*_g_ as a function of polymer swelling and state of charge. Data shown are for PMAP. Similar trends are observed for PEPP and PVBP (Figure S1). Polymer swelling is expressed as the electrolyte solution volume %. Lines connect the simulated points.

Figure 2 shows how *T*_g_ varies with increasing swelling of the polymer film and at different states of charge, for PMAP. We find that *T*_g_ decreases with increasing swelling of the polymer film. The decrease in *T*_g_ is expected as the swelling introduces (mainly) solvent molecules, which act as plasticizers. Figure 2 also shows that *T*_g_ decreases as the polymer gets charged. In this case, it is the TBA^+^ counterions that act as plasticizers. The *T*_g_ decrease caused by swelling is more marked for the uncharged polymers than for the charged ones—this is due to the fact that *T*_g_ for the charged polymer is already considerably lowered by the plasticizing effects of the TBA^+^ counterions. The same trends are found for the other polymers and are shown in Figure S1. In summary, for all polymers, *T*_g_ decreases with both increased swelling and increased state of charge.

### 2.3 Structural Characterization

We next analyze structural properties of the different polymers, first as a function of swelling, then as a function of state of charge. To mitigate the effects of different values of *T*_g_ (Figure 2) on the structural analysis of the different polymer systems, we perform the structural characterization at the temperatures *T* = 0.8 × *T*_g_ and *T* = 1.2 × *T*_g_. Since the findings from the *T* = 1.2 × *T*_g_ and *T* = 0.8 × *T*_g_ simulations are very similar, we will only discuss the structural characterization at *T* = 1.2 × *T*_g_ in the main text, and the *T* = 0.8 × *T*_g_ data can be found in the Supporting Information.

We first note that swelling has a negligible effect on the structure of the polymers as characterized by radial distribution functions (RDFs) (Figure S5). In particular, we look at the RDF between (the centroids of) phthalimide units, which gives us insights into phthalimide packing, and the RDF between (the centroids of) phthalimide units and TBA^+^ counterions, which informs us on the location of counterions with respect to the redox centers. The negligible impact of swelling on the polymer structures, as probed via the RDFs, applies to all polymers (Figure S5). We conclude that these polymers are structurally robust to the uptake of electrolyte solution, that is, we observe no structural changes such as different packing motifs for the phthalimides, for the swellings of up to 20% electrolyte solution volume investigated here.

**Figure 3:**
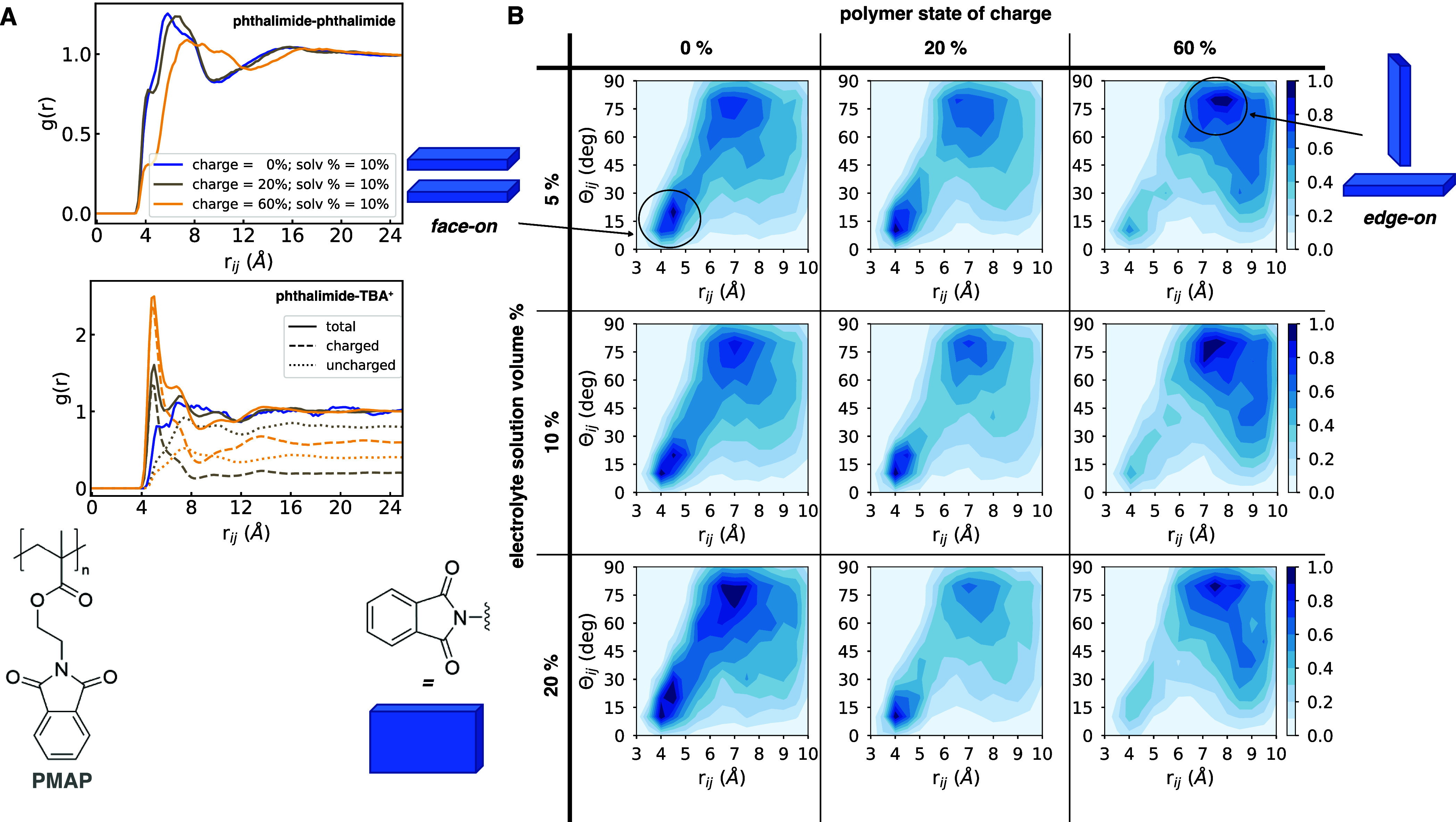
Structural characterization of the poly(methyl methacrylate)-based polymer. (A) RDFs between the centroids of phthalimide units (top) and between phthalimides and TBA^+^ ions (bottom); the dashed and dotted lines show the fraction of the RDF due to the *charged* and *uncharged* phthalimides, respectively. (B) Phthalimide pair configurational maps obtained by partitioning the pairs of a given condensed-phase in the two-dimensional space defined by the distance between two phthalimide units, *r*_*ij*_, and the angle between the vectors normal to the phthalimide planes, θ_*ij*_. Only pairs with *r*_*ij*_ ≤ 10 Å were considered. *T* = 1.2 × *T*_g_.

We next examine the same RDFs but now as a function of state of charge of the polymer. The phthalimide–phthalimide RDF in Figure 3A shows that, as the polymer goes to higher states of charge, there is a decrease in the first RDF peak at around 4 Å and a decrease (and shift toward larger distances) of the second RDF peak at around 7–8 Å between 20 and 60% state of charge. These decreases indicate a reduction in phthalimide–phthalimide interactions upon polymer charging. Simultaneously, there is a drastic increase in the first phthalimide–TBA^+^ RDF peak (Figure 3A, bottom), indicating an increase in phthalimide–TBA^+^ interactions upon polymer charging.

By analyzing the contributions to this RDF due to charged and uncharged PMAP monomers, we can see that the drastic increase in the first phthalimide–TBA^+^ RDF peak is almost entirely due to *charged* phthalimide–TBA^+^ interactions (dashed lines in Figure 3A), as could be anticipated. The effect of the change of state of charge on the structure that we just described for PMAP is very similar also in the case of PEPP and PVBP (see Figure S7), with the drastic change again happening between state of charge 20 and 60%. The only qualitative difference between the polymers is the significantly smaller increase in phthalimide–TBA^+^ interactions in the case of PEPP (Figure S7). The reduced interactions are due to the more limited space between phthalimides available to accommodate TBA^+^ ions, caused by the shorter backbone–phthalimide linker of PEPP with respect to PMAP and PVBP. Finally, in terms of the overall phthalimide–phthalimide RDF features, PEPP is qualitatively different from PMAP and PVBP (Figure S7), as discussed more in detail in the section below.

To get a better understanding of the solid-state packing underlying the RDFs as shown in Figure 3A, we analyze phthalimide–phthalimide pair configurations. We consider all the phthalimide–phthalimide pairs that are within a *r*_*ij*_ = 10 Å cutoff distance, where *i* and *j* are the indices of two phthalimide units and *r*_*ij*_ is the distance between their centroids. We subsequently compute other geometrical descriptors for such pairs, such as the angle between the vectors normal to the phthalimide planes, θ_*ij*_, and obtain two-dimensional maps such as the ones shown in Figure 3B (see Methods for further details). Such maps allow us to distinguish between different phthalimide packings, such as *face-on* (*r*_*ij*_ ≈ 4 Å and 0 ≤ θ_*ij*_ ≤ 25°) and *edge-on* (*r*_*ij*_ > 6 Å and 65 ≤ θ_*ij*_ ≤ 90°) stacking configurations, schematically shown in Figure 3B.

Figure 3B shows how configurational maps change as a function of swelling and polymer state of charge, for PMAP. First of all, the maps show how the peak around 4 Å in the phthalimide–phthalimide RDF of Figure 3A is due to face-on configurations, while the second peak is dominated by edge-on configurations. Moreover, the key observation that emerges from the maps is the almost complete disappearance of face-on configurations when going from a state of charge of 20 to 60%. This is consistent with, and sheds more light on, the RDF results previously discussed. Similar trends are observed for PEPP (Figure S9) and PVBP (Figure S10). However, PEPP shows a lower amount of face-on configurations to begin with, which explains the qualitative difference in the RDFs noted earlier. To summarize the findings from the configurational maps, the phthalimide orientational packing goes from face-on dominated to edge-on dominated as the polymers become more highly charged.

A final aspect of interest that we can probe from a structural point of view is whether phthalimide–phthalimide pairs are predominantly *intra*- or *inter*-polymer chain. Figure S12 shows that pairs are predominantly interchain, with such pairs constituting approximately 75, 70, and 70% of the total number of pairs in the case of PMAP, PEPP, and PVBP, respectively (at 5% swelling). Swelling again has an almost negligible impact, while the polymer state of charge reduces the amount of interchain pairs, especially so in the case of PEPP. At 60% charge, interchain pairs constitute approximately 65, 48, and 60% of the total number of pairs in the case of PMAP, PEPP, and PVBP, respectively. This is consistent with the fact that, due to TBA^+^ intercalation upon polymer charging, the spacing between polymer chains is expected to increase, thereby decreasing the likelihood of interchain pairs. In summary, the structural characterization shows that, upon polymer charging, TBA^+^ counterions interact with charged phthalimide units and by doing so disrupt face-on stacking between phthalimides.

### 2.4 Ionic Diffusivity

To provide insights into the ionic properties of this class of organic mixed ionic-electronic conductors, we next investigate ion diffusivity in our systems. To assess ion diffusion, we examine the mean squared displacement of TBA^+^. We note that these ions do not reach the diffusive regime over the time interval over which we computed the mean squared displacement (see Figure S14). However, we can assess trends between the different systems and gather insights on the effect that state of charge, swelling, and polymer backbone have on ion diffusivity by extracting diffusion coefficients for the longest simulated times.

**Figure 4:**
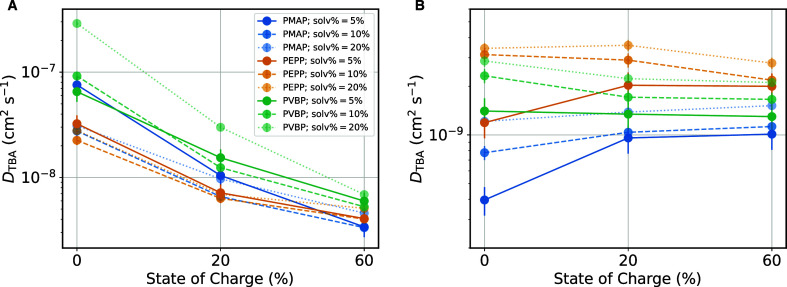
TBA^+^ ion diffusion coefficient, *D*_TBA_, as a function of state of charge and swelling for the different polymers. (A) *T* = 1.2 × *T*_g_ and (B) *T* = 300 K. The values of *D*_TBA_ are obtained using the Einstein relation from the mean squared displacement of TBA^+^ ions.

Figure 4 shows the diffusion coefficients of TBA^+^, *D*_TBA_, computed from the ion mean squared displacement using the Einstein relation (see also Figure S14), for the different polymers at different states of charge. To mitigate the effects of different *T*_g_’s (Figure 2), we first examine the behavior of the diffusion coefficients at *T* = 1.2 × *T*_g_ (Figure 4A). The three polymers all show a decrease in *D*_TBA_ by approximately 1 order of magnitude when their state of charge increases from 0 to 60%. We ascribe this to increased electrostatic interactions that slow down ion diffusion. Differences between the three polymers are minor, with PMAP and PVBP showing somewhat larger *D*_TBA_ values, which we ascribe to the higher temperatures (because of the higher *T*_g_ of these systems with respect to PEPP, Figure S1). In the Supporting Information, Figure S13, we plot *D*_TBA_ as a function of distance from *T*_g_ [in particular, 1000/(*T* – *T*_g_)] for the various systems. Consistently with Figure 4A, the plot shows that *D*_TBA_ coefficients generally increase with increasing swelling %, while they decrease with increasing state of charge.

We now also examine TBA^+^ diffusion coefficients at *T* = 300 K (Figure 4B), as these diffusion coefficients are expected to correlate with experimental ionic diffusivity during battery operation at room temperature. On Figure 4B, PEPP shows the largest TBA^+^ diffusion coefficients overall, reflecting PEPP’s backbone higher segmental mobility. The behavior of *D*_TBA_ at *T* = 300 K as a function of state of charge and swelling can be explained in terms of lowering of the systems’ *T*_g_. Overall, the poly(ethylene oxide) backbone provides the largest ion diffusion coefficients among the investigated phthalimide-containing polymers at room temperature, with *T*_g_ of the systems overall controlling *TBA*^+^ diffusivity.

### 2.5 Electronic Couplings

We now connect the larger length scales sampled by atomistic molecular dynamics (solid-state packing) to the molecular-level electronic picture, a connection that is lacking in the literature for this class of polymers with redox-active units other than TEMPO. In particular, we start from evaluating electronic couplings between phthalimide pairs. The electronic coupling has a strong impact on the kinetics of charge transport in these systems, which as mentioned above, generally transport via a charge-hopping mechanism.^1,21,22^ Such electronic couplings are greatly affected by the solid-state packing of the redox-active units.

Each of our condensed-phase systems contains between 5 × 10^3^ and 13 × 10^3^ phthalimide pairs that are within a 10 Å *r*_*ij*_ cutoff distance. To make the computation of electronic couplings for many such condensed-phase systems feasible, we use two approximations. First, we approximate the strength of the electronic coupling through evaluating orbital overlaps between the two molecules in the pair. This approximation, that is widely applied in the field for organic semiconductors,^32^ holds well also for these phthalimide-based polymers studied in this work, as can be seen by the linear correlation that we find between orbital overlaps and DIPRO (DImer PROjection)^33^ electronic couplings (see Methods for details) shown in Figure S16. A similar correlation between orbital overlaps and electronic couplings between redox-active groups has also been recently observed in ref (30). Second, we trained an artificial neural network as a surrogate model for orbital overlap calculations, achieving good accuracy (*R*^2^ = 0.83–0.92, Figure S17; with distribution means predicted with very good accuracy, Figure S18), and in this way further increasing the throughput. For details on the data set construction and training, see Methods.

**Figure 5:**
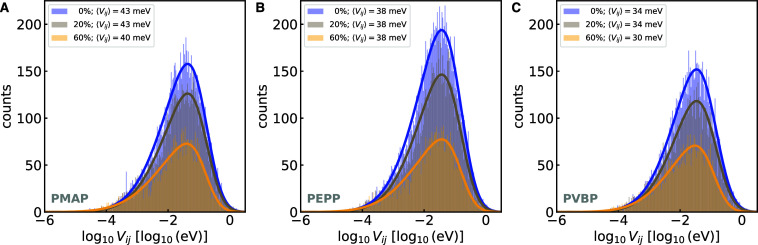
Electronic coupling distributions as a function of state of charge for the different polymers. (A) PMAP, (B) PEPP, and (C) PVBP. Mean electronic couplings, ⟨*V*_*ij*_⟩, obtained as the mode of the skew Gaussian distribution fits, are reported in the legends. Electrolyte solution volume % = 10%. *T* = 300 K.

Figure 5 shows how the distribution of electronic couplings changes as a function of polymer state of charge. For all polymers, we observe that (1) there is a clear decrease in total number of pairs within the 10 Å *r*_*ij*_ cutoff distance when going from 0 to 20 to 60% state of charge, (2) the peak of the distribution remains constant between 0 and 20% for all polymers, while (3) it shifts by 3–4 meV lower couplings between 20 and 60% for PMAP and PVBP but not for PEPP, suggesting a higher robustness of PEPP’s couplings to changes in state of charge, which we ascribe to the lower amount of face-on configurations observed earlier, and (3) PMAP shows the largest couplings, followed by PEPP and then PVBP. Together with the RDF results, these results show how the significant decreases in face-on configurations at 60% state of charge lead to a significant shift of the electronic coupling distribution that is likely detrimental to electron transport.

Another difference between the polymers that emerges from Figure 5 is the greater absolute number of pairs found for PEPP within the given cutoff distance. In particular, PEPP shows greater number of pairs within the cutoff distance with respect to the other polymers, especially at lower states of charge. The larger number of pairs is consistent with its expected higher density of redox sites enabled by the fewer number of “non-phthalimide atoms” of this polymer. Indeed, PEPP has the largest phthalimide density among the three polymers (Figure S19). This higher density of redox sites translates to a higher number of redox pairs as compared to PMAP and PVBP. We also note that all polymers with state of charge between 0 and 20% show a density of redox-active units in the range of 2.00–3.3 cm^–3^ (Figure S19), a range that is larger than the density estimated for the record-high conductivity polymer PTEO (estimated to be at least 1.75 cm^–3^) .^17^ Finally, the differences between the polymers in terms of both number of radical pairs and radical density are somewhat attenuated as the state of charge reaches 60% (Figures 5 and S19).

### 2.6 Electronic Percolation

While the electronic coupling distributions we have just seen provide a measure of the average strength of the electronic couplings between nearby redox units in the system, they are only part of the requirements for effective charge transport. Charge transport between sites that are (close to) immobile is essentially a percolation process. Hence, we now analyze electronic percolation in these systems.

We characterize electronic percolation by computing the Kirchhoff transport index, *K*_T_, a graph-theoretic metric that is useful to evaluate the overall resistance within an electronic network.^34^*K*_T_ uses a graph-based formulation of transport in which each graph *vertex* represents a radical site in the condensed-phase morphology (a localized charge transport state), and each graph *edge* represents the electronic coupling between such sites (see Methods for details). The larger *K*_T_ is, the less *resistive* (and hence more conductive) a network is to electronic percolation.

**Figure 6:**
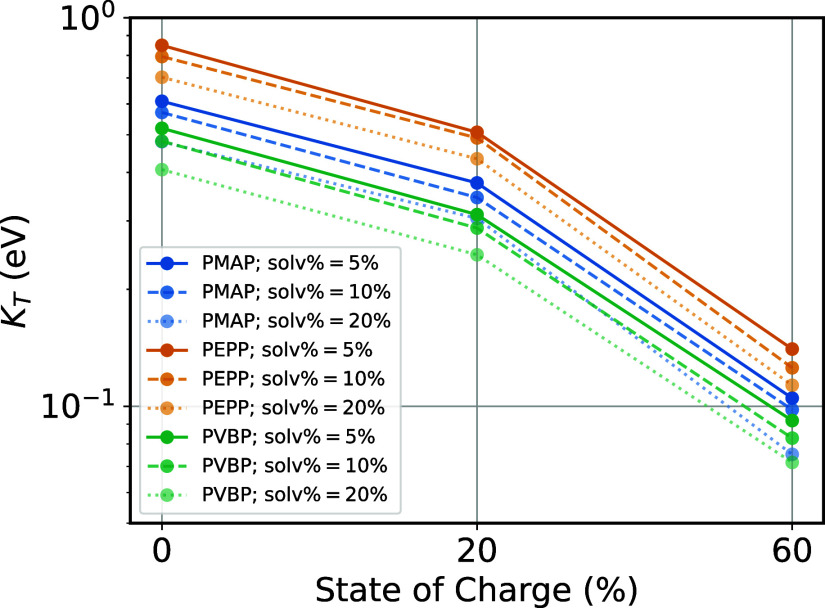
Electronic percolation as a function of state of charge and swelling. Computed Kirchhoff transport index, *K*_T_, for all the systems. *T* = 0.8 × *T*_g_. Electronic coupling threshold = 100 meV.

Figure 6 shows *K*_T_ for the various systems, from which we can draw the following conclusions. Echoing the structural results, swelling (up to 20%) has a minor impact on *K*_T_, with the expected effect of lowering *K*_T_. Consistently with the previous results, we see instead a large impact of the polymer state of charge: when going from 0 to 60% charged states, *K*_T_ drops by almost an order of magnitude in all cases. Finally, the hierarchy PEPP > PMAP > PVBP emerges between the different polymers, indicating that PEPP gives rise to the least resistive (i.e., most conductive) percolating networks among these polymers.

Comparing the electronic percolation results of Figure 6 to the phthalimide density noted previously (Figure S19), we note a very strong linear correlation between the two (Figure S20). This strong correlation is due to the coupling strengths being relatively similar between the different systems (Figure 5), leaving the density of radical sites to control *K*_T_.

### 2.7 Prediction of Experimental Observables

While the charge transport metric *K*_T_ is a useful computational metric, it is not an experimental observable. We thus now compute the predicted *apparent diffusion coefficient*, *D*_app_, an experimental observable that can be obtained via chronoamperometry or cyclic voltammetry.^21^*D*_app_ includes electron diffusion due to electron hopping between redox centers and the physical motion of redox centers; hence, in general *D*_app_ is given by^35^
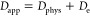
1where *D*_phys_ is the physical diffusion coefficient and *D*_e_ is the electron-hopping diffusion coefficient. If we assume that Laviron–Andrieux–Savéant theory^36,37^ holds, which is expected to apply for the glassy systems investigated here,^21^ then the apparent kinetics of electron transport is treated as “bounded diffusion” through redox-active pendant groups attached to an immobile backbone. Hence, we have *D*_phys_ = 0, and eq 1 reduces to^35^
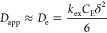
2in which *k*_ex_ is the bimolecular rate constant for electron self-exchange, *C*_E_ is the concentration of redox species, and δ is the center-to-center distance at electron transfer. *k*_ex_ is in general given by^35^
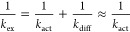
3where *k*_act_ is the bimolecular activation-limited rate constant for electron self-exchange, and following ref (35)., we assume mean-field conditions and hence neglect the contribution of diffusion (*k*_diff_ = 0). Electron hopping between nearby redox centers can be viewed as a Poisson process with the time constant *t*_hop_ representing the average time between electron hop attempts.^35^ If so, *t*_hop_ is related to *k*_act_ according to
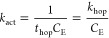
4where *k*_hop_ is the bimolecular hopping rate for electron self-exchange that can be computed via Marcus–Hush theory^38−40^
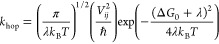
5in which *V*_*ij*_ is the electronic coupling between two redox sites, λ is the reorganization energy, Δ*G*_0_ is the standard free energy of the reaction, *k*_B_ is the Boltzmann constant, and *T* is the temperature. For our case, the case of self-exchange reactions, Δ*G*_0_ = 0.^41^ Using eqs 2–4, we arrive at the following, which we can use to estimate *D*_app_
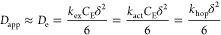
6

We can readily obtain all the other parameters needed for eq 6 from our simulations: *k*_hop_ from the mean of the electronic coupling distributions of Figure 5 and computed λ values (reported in Table S2; see also Methods) and δ as an electronic coupling-weighted mean distance (δ = ∑_*n*_*V*_*n*_*x*_*n*_/∑_*n*_*V*_*n*_, where *V*_*n*_ is the electronic coupling *V*_*ij*_ and *x*_*n*_ is the *r*_*ij*_ distance for the *n*-th phthalimide pair; see Figures S24–S26 for plots of the raw data).

**Figure 7:**
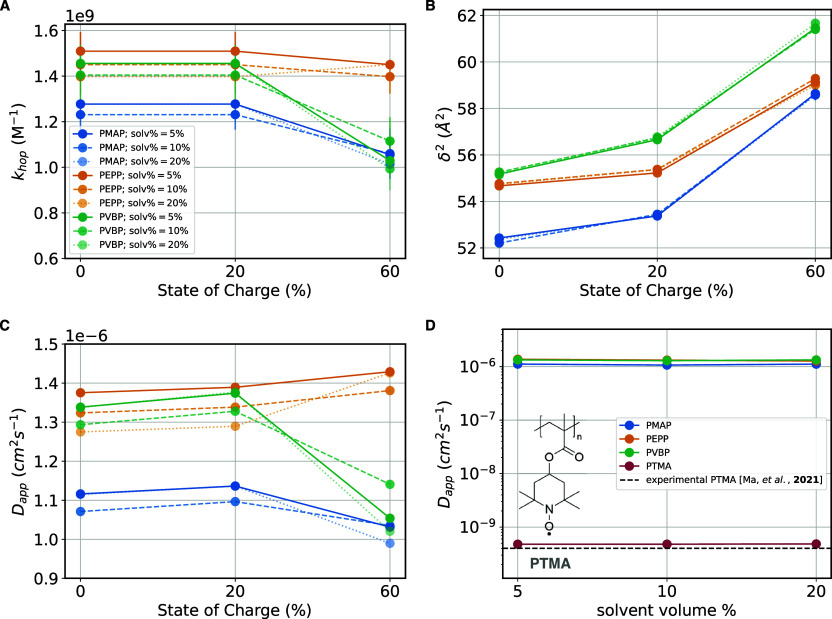
Predicted electron transport performance of systems investigated. (A) Electron self-exchange rate constants (*k*_hop_), (B) squared center-to-center distances at electron transfer (δ^2^), and (C) the resulting predicted Laviron–Andrieux–Savéant apparent diffusion coefficients (*D*_app_, via eq 6). (D) Comparison of predictions of *D*_app_ for the phthalimide-containing polymers and for a reference nitroxide radical-based polymer PTMA and the available experimental data for PTMA from ref (42).

The *k*_hop_ and δ parameters needed to compute *D*_app_ are shown in Figure 7A–C for all the systems, along with the resulting *D*_app_. We note the following: (1) PEPP and PVBP show the largest *k*_hop_ values; PVBP’s relatively low electronic coupling strengths (Figure 5) are compensated by PVBP monomer’s showing the lowest inner λ among the polymers (Figure S27); instead, PMAP monomer’s largest inner λ among the three backbones offsets the effect of PMAP’s highest electronic coupling strengths (Figure 5); (2) *k*_hop_ values for PMAP and PVBP decrease when going from 20 and 60% state of charge, while they remain constant for PEPP, following the behavior of electronic coupling distributions (Figure 5); (3) δ values are smallest for PMAP, which is thus found to be able to pack phthalimide units at the shortest distances, even surpassing PEPP despite PEPP’s largest density of radical sites (Figure S19); this is likely due to the short backbone–phthalimide linker in PEPP not allowing for phthalimide units to pack as good as in the case of PMAP, which corroborates the observed lower amount of face-on configurations in PEPP; (4) PVBP shows the largest δ, which is consistent with PVBP possessing the bulkiest monomer among the investigated polymers; importantly, given that these larger δ values are not accompanied by large decreases in *V*_*ij*_ (Figure 5), large δ values are a positive feature in terms of electronic transport for PVBP because they allow this polymer to effectively transport electrons over larger distances with each hopping step, hence favoring *D*_app_; (5) the impact of the polymer state of charge echoes what we have seen so far by overall leading to larger δ and lower *k*_hop_ and consequently lower *D*_app_ values; however, the effects of the state of charge on δ and *k*_hop_ compensate each other and result in a decrease of the impact of the state of charge on *D*_app_; we note that experimentally *D*_app_ at different states of charge cannot be probed; and solvent swelling has, again, a small impact overall; and (6) these factors combined via eq 2 results in the following *D*_app_ ranking: PEPP > PVBP > PMAP. In summary, the low inner reorganization energy (molecular-level property) and relatively large effective electron-hopping distance (condensed-phase property) rank PVBP unexpectedly high in terms of electron transport capabilities, while the robustness to structural disorder (introduced by state of charge) and a good balance of inner reorganization energy and electronic coupling strength make PEPP the polymer with the expected largest electron transport capabilities.

We note that the predicted *D*_app_ (on the order of 10^–6^ cm^2^ s^–1^, Figure 7) are larger than literature values for the well-studied poly(TEMPO methacrylate), PTMA, system in the solid state (≈10^–10^ cm^2^ s^–1^, see Table S3) .^20,24,42,43^ Since experimental data on the newly proposed polymers are unavailable, to validate our computational approach we computed *D*_app_ for a PTMA-based solid-state system for which detailed experimental data are available.^42^ In particular, Ma et al. measured a *D*_app_ of 4.0 × 10^–10^ cm^2^ s^–1^ for the PTMA in H_2_O/TEABF_4_ at a nitroxide radical concentration of 3.7 M (Table S3) .^42^ We model this PTMA in H_2_O/TEABF_4_ system with the same protocol used for the phthalimide-containing polymers (see Figure S29 and Table S4; we note that the 3.7 M concentration corresponds to a swelling of about 10%). For swellings between 5 and 20%, we obtain a *D*_app_ of 4.8 × 10^–10^ cm^2^ s^–1^, which falls close to the experimental value (Figure 7D). This result validates the multiscale model developed in this work to connect the molecular to the macroscopic picture for the prediction of electronic properties of this class of RAPs.

Comparing the predicted *D*_app_ values for the phthalimide-based polymers to the predicted *D*_app_ value for PTMA, we predict an increase of more than 3 orders of magnitude (Figure 7D). The larger *D*_app_ values for the phthalimide polymers are due to predicted *k*_hop_ values (≈10^9^ s^–1^) that are larger than PTMA’s (≈10^5^ s^–1^, Table S4). The *k*_hop_ is controlled by the values of λ and *V*_*ij*_, both of which are much improved in the phthalimide-containing polymers proposed here. Regarding λ, as already reported,^27^ TEMPO exhibits an anomalously high inner reorganization energy, and unsurprisingly, this holds true also for the PTMA monomer (λ_in_ = 0.993 eV), which is about 65% larger than any of the λ_in_ of the phthalimide-containing monomers, see Table S2. Regarding *V*_*ij*_, PTMA exhibits mean electronic couplings of 8 meV in the solid state (Figure S29C), a value which is 4–5 times smaller than the values we find for the phthalimide-containing polymers (43–30 meV, Figure 5). Accordingly, the relatively high electronic couplings that the phthalimide-containing polymers are found to achieve in the solid-state combined with a relatively low inner reorganization energy are responsible for the very high electron transport capabilities predicted here.

## 3 Conclusions

We computationally investigated structural, ionic, and electronic properties of phthalimide-based polymers for applications as solid-state n-type materials for all-organic batteries. We assessed the impact of the polymer state of charge, swelling, and backbone chemistry on such solid-state properties. Simulations revealed that the polymer state of charge significantly affects the material’s structural, ionic, and electronic properties. While structural properties are robust to swellings up to 20% electrolyte solution volume, an increase in the polymer state of charge leads to a decrease in phthalimide–phthalimide face-on configurations, which in turn decreases electronic couplings. Increases in state of charge and swelling are both found to increase ionic diffusivity due to the decrease in the systems’ *T*_g_ they imply. Importantly, by combining information from several length scales, our multiscale approach allows us to bridge the gap between bottom-up molecular characteristics and macroscopic properties such as the *D*_app_. Prediction of *D*_app_ for the well-studied, reference polymer PTMA allows us to validate our approach, which we then use to rank the phthalimide-containing polymers based on their electron transport capabilities. The low inner reorganization energy (molecular-level property) and relatively large effective electron-hopping distance (condensed-phase property) rank the polystyrene-based polymer PVBP unexpectedly high in terms of electron transport capabilities, while the robustness to structural disorder (introduced by state of charge) and a good balance of inner reorganization energy and electronic coupling strength achieved in the solid state make the poly(ethylene oxide)-based polymer PEPP the one with the predicted largest electron transport capabilities. Overall, due to high electronic couplings achieved in the solid state and relatively low inner reorganization energies, the investigated phthalimide-containing polymers are found to be very promising electron transport materials, with predicted *D*_app_ on the order of 10^–6^ cm^2^ s^–1^, which is 3 orders of magnitude larger than *D*_app_ coefficients for the reference, nitroxide radical-based polymer PTMA.

The multiscale approach developed herein allows for atomistically detailed computational investigations of RAP candidates. As such, the protocol can be used to probe the impact of different electrolytes (which, for example, have been recently found to affect the material capacity by as much as 1000%^31^), different or differently substituted redox units, and other molecular engineering targets, delivering predictions of experimental observables such as *D*_app_. Future work will also include extension to coarse-grained scales by resorting to recently developed electronic coarse-grained modeling schemes,^44,45^ opening the way to the study and prediction of processes at large spatiotemporal scales, such as molecular diffusion during cyclic voltammetry, and electronic transport over large-scale molecularly detailed samples.

## 4 Methods

### 4.1 Atomistic Models

All polymers contain 30 repeat units and are terminated by methyl groups. Initial OPLS-AA/CM1A force field parameters for the polymers and the DME solvent were obtained from LigParGen.^46,47^ We use 1.20 × *q*_CM5_ charges,^48^ that are compatible with OPLS-AA as validated in ref (49). to model the excess charge gained by each phthalimide unit upon reduction. In particular, each charge in a charged phthalimide monomer is computed as follows: *q*_*i*_^red^ = *q*_*i*_^CM1A,neu^ + (*q*_*i*_^CM5,red^ – *q*_*i*_^CM5,neu^). For PTMA, we used an OPLS-AA/CM1A-based force field from our previous work,^44^ available via Polyply.^50^ We used the TIP3P^51^ water model for the swollen PTMA simulations. We modeled TBA^+^, PF_6_–, TEA^+^, and BF_4_– via OPLS-AA-based models available in the literature.^52,53^ Following the recent systematic study of Doherty et al.,^53^ we used a molecular charge scaling factor of 0.8 × *q* for all the *charged* species (cations, anions, and the charged monomers within a polymer), an effective approach that has recently emerged to correct for the polarization and charge transfer effects that are missing in fixed-charge atomistic force fields.^53^ Topologies for all the polymers were built via Polyply.^50^ The charged polymer sequences are generated randomly using the polyply gen_seq tool^50^ first, that generates a JSON file with a random sequence for each of the 100 chains; and those files are then used to generate the polymer topology file via polyply gen_params.

### 4.2 Molecular Dynamics Simulations

Starting configurations for all systems were prepared via Polyply.^50^ All 27 systems (3× polymers, 9× swelling, and state of charge conditions) were equilibrated at 900 K for at least 100 ns—a time that allowed for polymer chain relaxation, as assessed by determining when the end-to-end vector autocorrelation function reached a value below 0.1. Snapshots extracted after 100, 150, and 200 ns at 900 K were then cooled down to 100 K (cooling rate = 10 K/ns). Density data obtained during the cooling simulations were used to determine the glass-transition temperature, *T*_g_, by fitting the low-temperature (glassy) regime and high-temperature (melt) regime linearly as described in detail in the Supporting Information. The obtained *T*_g_ values are shown in Figure S1. From the cooling trajectories, snapshots at *T*_g_ = 0.8 × *T*_g_, *T*_g_ = 1.2 × *T*_g_, and 300 K were extracted and relaxed further at those temperature for 100 ns. We used the Verlet scheme with a nonbonded cutoff of 1.1 nm, dispersion corrections, and the particle mesh Ewald method for long-range electrostatics. A time step of 1 fs was used while bonds involving hydrogens were constrained via the LINCS algorithm. Temperature and pressure control was done via a Nosé–Hoover thermostat and a Parrinello–Rahman barostat (coupling parameters, τ_P_, of 1 and 5 ps, respectively); and a Berendsen barostat was used for equilibration purposes (τ_P_ = 1 ps). All molecular dynamics simulations were run with Gromacs version 2021.x or higher.^54^

### 4.3 Molecular Configuration Analysis

The configurational maps shown in Figure 3B (and Figures S9, S10) were obtained with the following steps: (i) phthalimide pairs were collected if *r*_*ij*_ ≤ 10 Å; (ii) for the selected pairs, other geometrical descriptors (e.g., θ_*ij*_), were computed; (iii) two-dimensional maps were computed by binning the pairs according to their (*r*_*ij*_, θ_*ij*_) values; and (iv) the maps were normalized by the volume and the number of *ij* pairs (see Figure S11). The procedure is implemented in custom Python scripts that make use of the MDAnalysis library.^55,56^

### 4.4 Electronic Structure Calculations

#### 4.4.1 Electronic Coupling Calculations

We used orbital overlaps as a proxy for the electronic couplings.^32^ We computed orbital overlaps as done in our recent work^44^ via Gaussian16 (version C.01)^57^ and Multiwfn^58^ ωB97X-D/def2-SV(P) level of theory. Orbital overlaps were computed between the least unoccupied molecular orbital (LUMO) of the neutral species and the singly occupied molecular orbital (SOMO) of the radical anion in the case of *N*-methyl-phthalimide ⟨ϕ_LUMO_|ϕ_SOMO_⟩ and the SOMO of the neutral radical and the LUMO of the cation, ⟨ϕ_SOMO_|ϕ_LUMO_⟩ in the case of TEMPO. Reference electronic couplings were computed with the DImer PROjection (DIPRO) method^33^ as implemented in CATNIP^59^ (a code recently used for mixed ionic-electronic conductors^60^). The same orbitals as for the orbital overlap calculations were used.

#### 4.4.2 Reorganization Energy Calculations

All the calculations were performed using Gaussian16 (version C.01)^57^ at the ωB97X-D/def2-SV(P) level of theory with the implicit DME solvent using the polarizable continuum model. The reorganization energy, λ, can be expressed as the sum of inner and outer reorganization energy, λ_in_ and λ_out_, separately. With Nelsen’s four-point method,^61^ λ_in_ can be estimated via
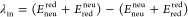
7where *E* represents the single-point energy of a molecule calculated at a redox state specified in superscript under the geometry optimized at a redox state specified in subscript. λ_out_ can be further estimated based on the simplified model given by Marcus^38^
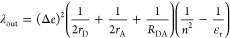
8where Δ*e* is the amount charge being transferred (Δ*e* = 1), *r*_D_ and *r*_A_ are the respective donor and acceptor radii, respectively, *R*_DA_ is the value of donor/acceptor separation, and *n* and ε_r_ are the refractive index and the static dielectric constant, respectively. ε_r_ is determined as a mixture of the dielectric constant of the polymer and the dielectric constant of the solvent as shown in Figure S27. Donor and acceptor radii, being same for the phthalimide unit, were estimated from the Universal Force Field radii using Gaussian16 (namely, *r*_D_ = *r*_A_ = 3.740 Å for *N*-methyl-phthalimide and *r*_D_ = *r*_A_ = 3.942 for TEMPO). We estimated *R*_DA_ by assuming it equals *r*_D_ + *r*_A_ at the closest approach. Note that eq 8 is in atomic units.

### 4.5 Surrogate Machine Learning Models

#### 4.5.1 Data Sets

Training data for the machine learning models (neural networks, see below) were generated with a procedure similar to our previous work^44^ but using a condensed phase of monomers (*N*-methyl-phthalimide or TEMPO) instead of the polymers. Namely, we sampled phthalimide pair configurations from a condensed phase simulation of pure phthalimide sampled at temperatures between 600 and 300 K; we extract ≈15, at intervals of 10 K between the two temperatures, and from each snapshot we extract ≈1000 pairs, leading to a total data set size of over 146 343 data points. The same was done for TEMPO, leading to a total data set size of 147 804 data points in that case.

#### 4.5.2 Input Representation, Model, and Training Details

As done in our previous work,^44^ we trained feed-forward artificial neural networks using the conformations (in the form of a reciprocal distance matrix) as input and the orbital overlaps as labels. In particular, for each pair conformation a reciprocal distance matrix **D** between all atoms was computed. Its elements are  where **r** is the position vector, *i* and *j* are the monomer indices, and *k* and *l* are the atom indices. Each matrix was flattened and the resulting one-dimensional vector (of dimension *N*^2^, with *N* = 12 for *N*-methyl-phthalimide and *N* = 11 for TEMPO) was used as the input feature for the supervised machine learning task. The base 10 logarithm of ⟨ϕ_SOMO_|ϕ_LUMO_⟩ was employed to assess orbital overlaps. To ensure flexibility, a fully connected, feed-forward neural network was utilized, comprising an input layer of dimension *M*, followed by four batch-normalized hidden layers, each with the same number of neurons. *M* is the dimension of the flattened input vector and is hence equal to *N*^2^. Hyperparameters, including the number of neurons in hidden layers, batch size, and training epochs, were optimized. Training utilized the default learning rate of the NAdam optimizer (0.001) and standard scaling was applied to input and output features. 10% of each data set was reserved for testing (holdout data set), while the remaining data points were used for training and validation through 5-fold cross-validation. Hyperparameters were fine-tuned via grid search using 5-fold cross-validated performance. The model’s final performance was evaluated by applying the best-performing model, as determined by 5-fold cross-validation, to the held-out test set. The results on the test set are shown on Figure S17. The surrogate models were implemented using the Keras^62^ and scikit-learn^63^ libraries.

### 4.6 Graph-Theoretic Approach to Percolation

To quantify electronic percolation capabilities of the systems, we use the Kirchhoff transport index, *K*_T_.^34^*K*_T_ corresponds to the summation of inverse resistances of all paths between any two points in a graph that represents the charge transport network, normalized by the total number of pathways . *K*_T_ is computed as follows: we first computed the weighted adjacency matrix, **A**, with elements
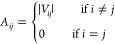
9was computed for each morphology, where *V*_*ij*_ is the electronic coupling between sites *i* and *j*. **A** is used to construct the admittance matrix, **Λ**, whose elements, Λ_*ij*_, correspond to the admittance between each site, that is, the inverse of the effective resistance. For more details, we refer to the original *K*_T_ work.^34^*K*_T_ is then finally calculated as
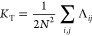
10in which we note that, to avoid double counting of Λ_*ij*_, we normalize by 2*N*^2^ (instead of *N*^2^ as done in ref (34)). We use a threshold of 100 meV to define if a pair is coupled or not in Figure 6. We note however that the analysis is very robust to different values of this threshold, as shown in Figure S21. Moreover, Figures S22, S23 show the number of networks and network sizes as a function of the threshold, respectively. We use the kugupu Python package to compute *K*_T_.^64^

## Data Availability

Models, code, and data associated with this work are available at https://github.com/ricalessandri/redox-active-polymers. All polymer models have been implemented in the Polyply^50^ library and are available at https://github.com/marrink-lab/polyply_1.0. Simulation workflows and data generated will be made available via CRIPT.^65^

## Supporting Information Available

*T*_g_ calculation details and additional *T*_g_ results; number of molecules contained in each system; schematic of the atomistic molecular dynamics protocol; additional RDFs and configurational maps; intramolecular vs intramolecular pair breakdown; electronic coupling correlations (overlap vs electronic couplings and DFT overlaps vs neural network-predicted overlaps); additional electronic percolation results; phthalimide interdistance vs overlap heatmaps; reorganization energies; and additional *D*_app_ results (PDF)
