# Supplementary material for: Structural, Ionic, and Electronic Properties of Solid-State Phthalimide-Containing Polymers for All-Organic Batteries
Source: JACS Au. 2024 Jun 7;4(6):2300–11. doi: 10.1021/jacsau.4c00276 (PMC11200234; doi:10.1021/jacsau.4c00276)
Supplement: Supplementary file 1 — au4c00276_si_001.pdf [file au4c00276_si_001.pdf]

Supporting Information:

Structural, Ionic, and Electronic Properties of Solid-State Phthalimide-Containing Polymers for All-Organic Batteries

Riccardo Alessandri,[†] Cheng-Han Li,[‡] Sheila Keating,[¶] Khirabdhi T. Mohanty,[§] Aaron Peng,[†] Jodie L. Lutkenhaus,[‖] Stuart J. Rowan,[†,¶] Daniel P. Tabor,[*,‡] and Juan J. de Pablo[*,†]

†*Pritzker School of Molecular Engineering, University of Chicago, Chicago, Illinois 60637, USA*

‡*Department of Chemistry, Texas A&M University, College Station, Texas 77842, USA*

¶*Department of Chemistry, University of Chicago, Chicago, Illinois 60637, USA*

§*Artie McFerrin Department of Chemical Engineering, Texas A&M University, College Station, Texas 77843, USA*

‖*Artie McFerrin Department of Chemical Engineering and Department of Materials Science & Engineering, Texas A&M University, College Station, Texas 77843, USA*

E-mail: daniel_tabor@tamu.edu; depablo@uchicago.edu

Contents

1 Glass Transition Temperatures **S-3**

2 Polymer-Electrolyte Systems **S-5**

3 Atomistic Molecular Dynamics Protocol **S-7**

4 Structural Properties **S-8**

5 Ionic Diffusivity **S-12**

6 Electronic Couplings **S-14**

6.1 Orbital overlaps *vs.* electronic couplings S-14

6.2 Neural network surrogate model for orbital overlaps S-15

7 Electronic Percolation **S-16**

8 Connection to Experimental Observables **S-20**

8.1 Phthalimide-phthalimide distance at electron transfer, δ (PMAP) S-20

8.2 Phthalimide-phthalimide distance at electron transfer, δ (PEPP) S-21

8.3 Phthalimide-phthalimide distance at electron transfer, δ (PVBP) S-22

8.4 Reorganizatione energies S-23

8.5 Additional results for the apparent diffusion coefficient, D_{app} S-24

8.6 Apparent diffusion coefficient (D_{app}) of PTMA S-25

References **S-26**

1 Glass Transition Temperatures

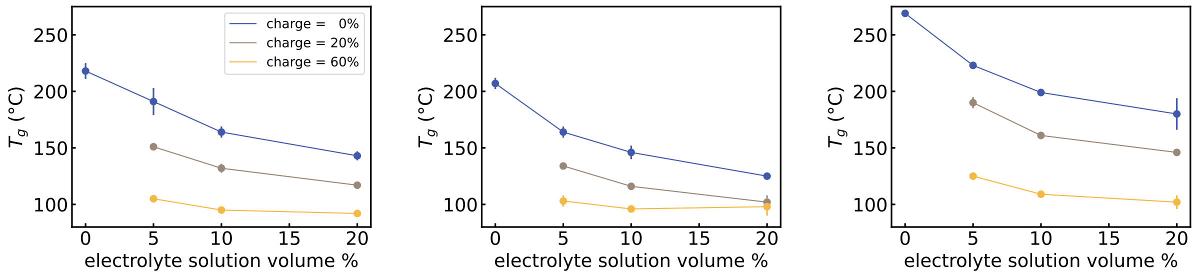

Figure S1: **Computed T_g versus electrolyte solution volume % and state of charge**. (left) PMAP; (center) PEPP; (right) PVBP.

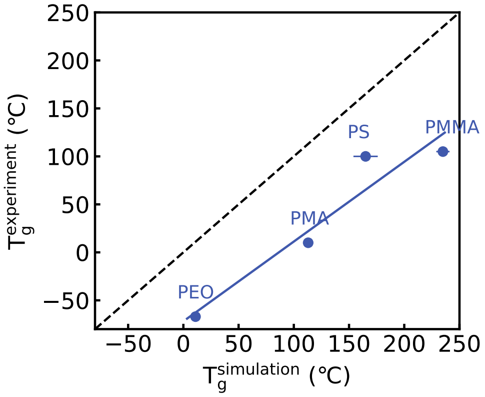

Figure S2: **Computed T_g *vs.* experimental T_g for the polymer backbones**. Names of the polymers are indicated on the datapoints (PMMA = polymethyl methacrylate; PEO = polyethylene oxide; PS = polystyrene; PMA = polymethyl acrylate). The blue line is the linear fit $Tg_g^{exp} = 0.83Tg_g^{sim} - 71.8$ ($R^2 = 0.92$).

To determine T_g, we used the same procedure as in Ref. S1. Namely, we fit the density-temperature data via two independent linear fits to find the melt and glass regions. To select the best fits, we did the following: starting from a glass fit starting from the fourth datapoint from the lowest T and a melt fit starting from the fourth datapoint from the highest T, we scanned through all combinations of glass and melt fits obtained by progressively increasing the number of datapoints to be included in the fits. The best fits were selected by choosing the ones that maximize the sum of the R^2 coefficients of the linear fits; T_g was then obtained as temperature at which the two linear fits cross (*e.g.*,, see Figure S3).

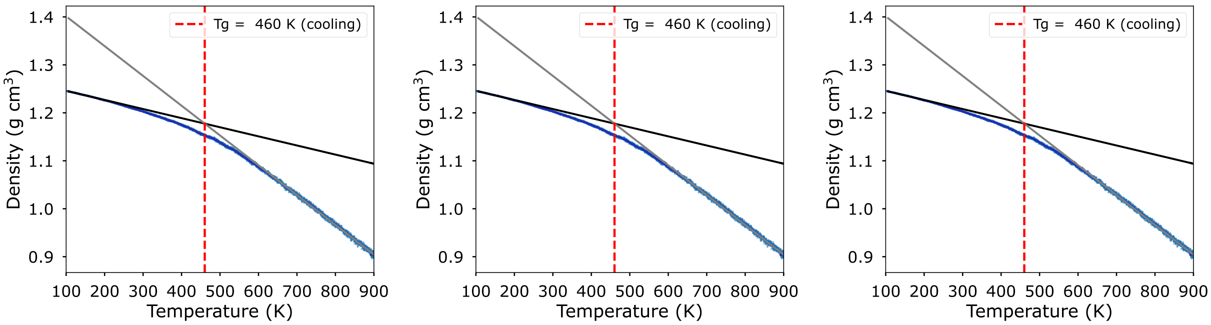

Figure S3: **Example T_g fits**. These three cooling trajectories are for the PMAP system at 0% state of charge and 5% swelling.

2 Polymer-Electrolyte Systems

Molecular composition of the systems. Table S1 reports all the molecules contained in the systems represented in Figure 1 of the main text for the various polymers.

Table S1: **Details for the various systems.** Number of solvent molecules and ions that compose each system. S.o.C. stands for "state of charge".

PMAP:DME:TBAPF$_6$ systems

**volume % / S.o.C.**	**0%**	**20%**	**60%**
**5%**	252× DME<br>17× TBA^+<br>17× $PF_6{}^-$	0% +600× TBA^+	0% +1800× TBA^+
**10%**	504× DME<br>34× TBA^+<br>34× $PF_6{}^-$	0% +600× TBA^+	0% +1800× TBA^+
**20%**	1008× DME<br>68× TBA^+<br>68× $PF_6{}^-$	0% +600× TBA^+	0% +1800× TBA^+

PEPP:DME:TBAPF$_6$ systems

**5%**	197× DME<br>13× TBA^+<br>13× $PF_6{}^-$	0% +600× TBA^+	0% +1800× TBA^+
**10%**	395× DME<br>27× TBA^+<br>27× $PF_6{}^-$	0% +600× TBA^+	0% +1800× TBA^+
**20%**	790× DME<br>53× TBA^+<br>53× $PF_6{}^-$	0% +600× TBA^+	0% +1800× TBA^+

PVBP:DME:TBAPF$_6$ systems

**5%**	274× DME<br>18× TBA^+<br>18× $PF_6{}^-$	0% +600× TBA^+	0% +1800× TBA^+
**10%**	548× DME<br>37× TBA^+<br>37× $PF_6{}^-$	0% +600× TBA^+	0% +1800× TBA^+
**20%**	1095× DME<br>74× TBA^+<br>74× $PF_6{}^-$	0% +600× TBA^+	0% +1800× TBA^+

Procedure to determine the composition of the systems at different swelling

ratios. We determined the composition of the systems based on the pure systems, the dry polymer and the electrolyte solution. We prepare a solution of 0.5 M by computing the number of ions needed for that concentration in a given volume, then using the Gromacs tool `gmx insert-molecules` to fill the given volume with solvent molecules. We then perform a short (2 ns) equilibration that leads to a negligible box volume adjustment. In parallel, we equilibrate the pure polymer systems, i.e., systems containing only 100 30-mer chains of the polymer, following the same protocol as shown in Figure S4. Based on the volume of the equilibrated pure polymers, we compute the volume that corresponds to a 5%, 10%, or 20% increase in volume. The computed volume increase is the volume of electrolyte solution that we add to realize the 5%-, 10%-, or 20%-swollen systems, respectively. What one actually needs is the number of molecules of electrolyte solution that corresponds to the 5%, 10%, or 20% volume. For example, the pure DME:TBAPF6 electrolyte solution that we equilibrated contains 1037 DME molecules, 70 TBA^+, and 70 PF_6^- ions. Meanwhile, an equilibrated box of 100 30-mers of PEPP has a volume of ≈843 nm^3; 5% of this volume is ≈42 nm^3. Given that the solvent box volume is ≈220 nm^3, ≈42 nm^3 of it will contain:

$$N_{\mathrm{DME},5\%} = 1037 \cdot \frac{42}{220} \approx 197$$
$$N_{\mathrm{TBA},5\%} = N_{\mathrm{PF6},5\%} = 70 \cdot \frac{42}{220} \approx 13$$

which are the numbers that are reported in Table S1 for the PEPP:DME:$TBAPF_6$ system. We use this composition—100 30-mers, 197 DME molecules, 13 TBA^+, and 13 PF_6^-—and run the protocol of Figure S4. The final volume of the equilibrated PEPP:DME:$TBAPF_6$ system is found to be ≈875 nm^3. This means that the effective swelling for the system with respect to the dry polymer is ≈4%, and that the system shows negative deviation from ideal mixing behavior. The effective swellings for PEPP's 10%- and 20%-swollen systems are found to be ≈8% and ≈16%, respectively. For all the systems the effective swellings are in the range 3.5-4%, 7-8%, and 14-16%, for the 5%, 10%, and 20% cases, respectively.

3 Atomistic Molecular Dynamics Protocol

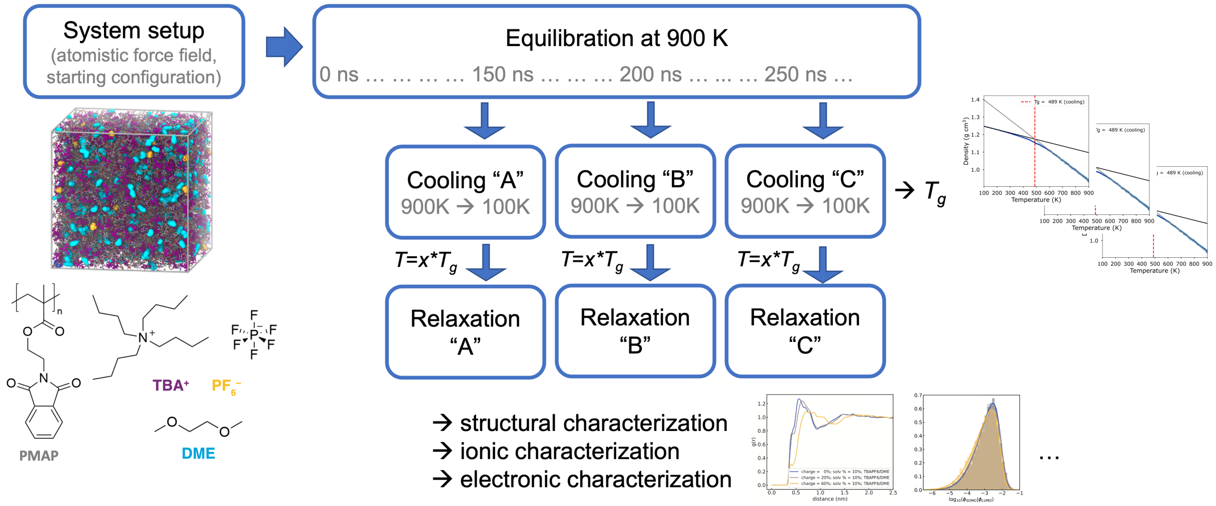

Figure S4: **Atomistic MD simulation protocol.** After the system is setup with Polyply,[S2] we equilibrate it well in the melt regime (900 K), after which configurations are taken out starting from 150 ns every 50 ns. These configurations are cooled down to 100K with a 10 K/ns rate. From the cooling trajectory, snapshots are taken at temperatures $T = 0.8 \cdot T_\mathrm{g}$, $T = 1.2 \cdot T_\mathrm{g}$, and $T = 300$ K that are relaxed at those temperatures for 100 ns. Strucutral, ionic, and electronic characterizations are conducted on the last 50 ns of the “relaxation” trajectories.

4 Structural Properties

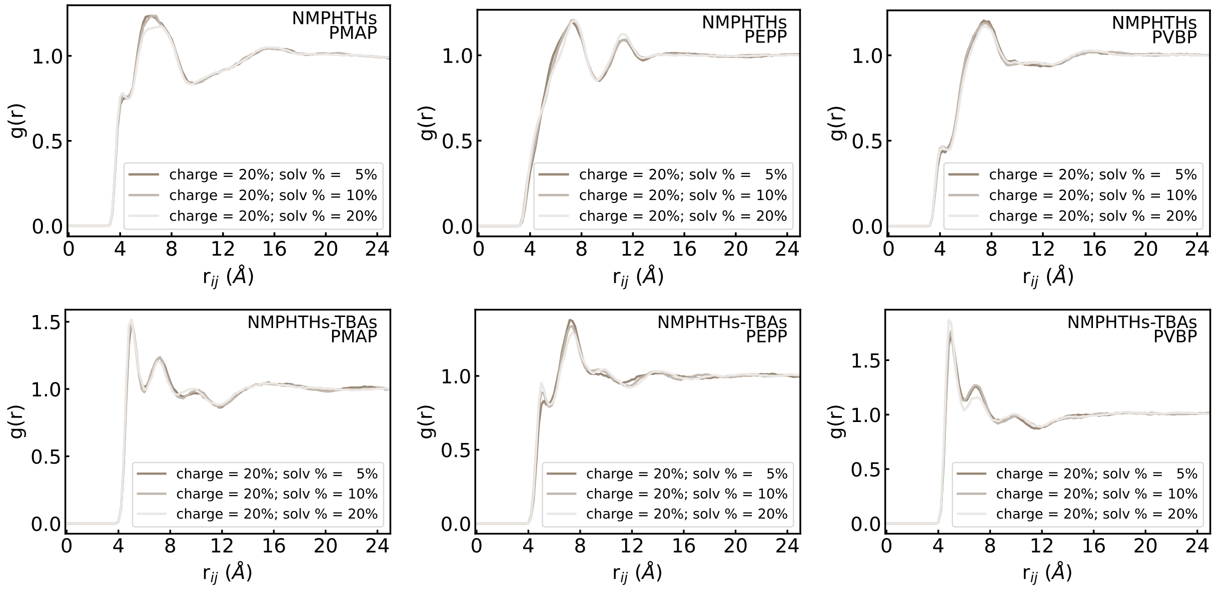

Figure S5: **RDFs for the polymers at $T = 1.2 \cdot T_g$ and state of charge = 20% at different swelling %.** Phthalimide-phthalimide (top row) and phthalimide-TBA$^+$ (bottom row) RDFs as a function of swelling % for the polymers: PMAP (left column), PEPP (central), and PVBP (right).

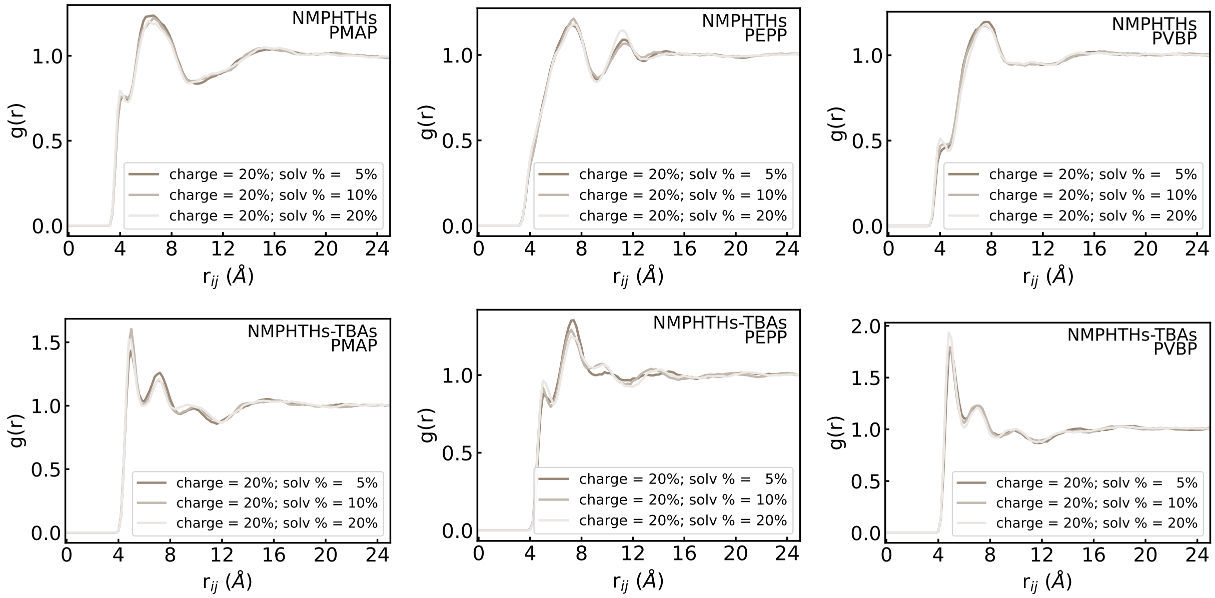

Figure S6: **RDFs for the polymers at $T = 0.8 \cdot T_g$ and state of charge = 20% at different swelling %.** Phthalimide-phthalimide (top row) and phthalimide-TBA$^+$ (bottom row) RDFs as a function of swelling % for the polymers: PMAP (left column), PEPP (central), and PVBP (right).

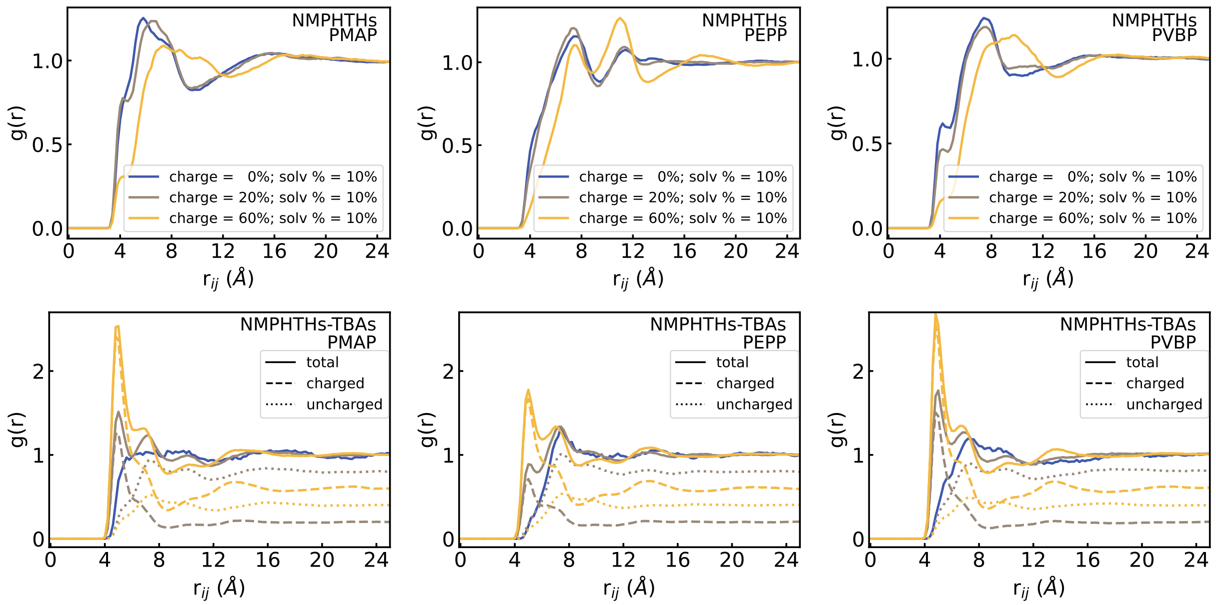

Figure S7: **RDFs for the polymers at $T = 1.2 \cdot T_\mathrm{g}$ and swelling = 10% at different states of charge.** Phthalimide-phthalimide (top row) and phthalimide-TBA$^+$ (bottom row) RDFs as a function of state of charge for the polymers: PMAP (left column), PEPP (central), and PVBP (right).

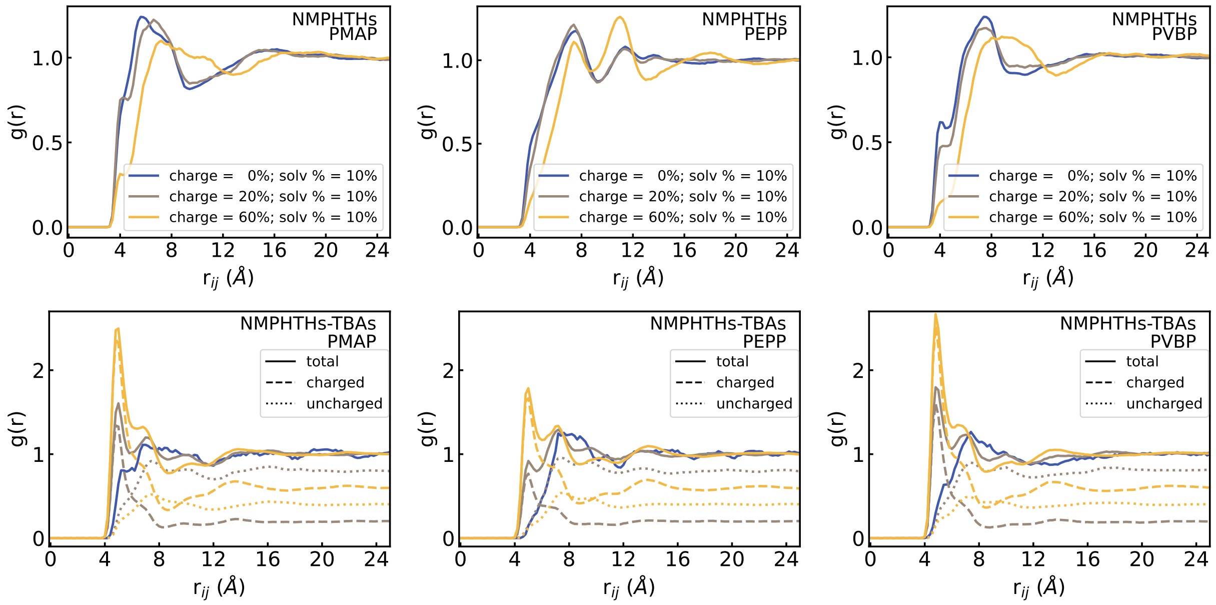

Figure S8: **RDFs for the polymers at $T = 0.8 \cdot T_\mathrm{g}$ and swelling = 10% at different states of charge.** Phthalimide-phthalimide (top row) and phthalimide-TBA$^+$ (bottom row) RDFs as a function of state of charge for the polymers: PMAP (left column), PEPP (central), and PVBP (right).

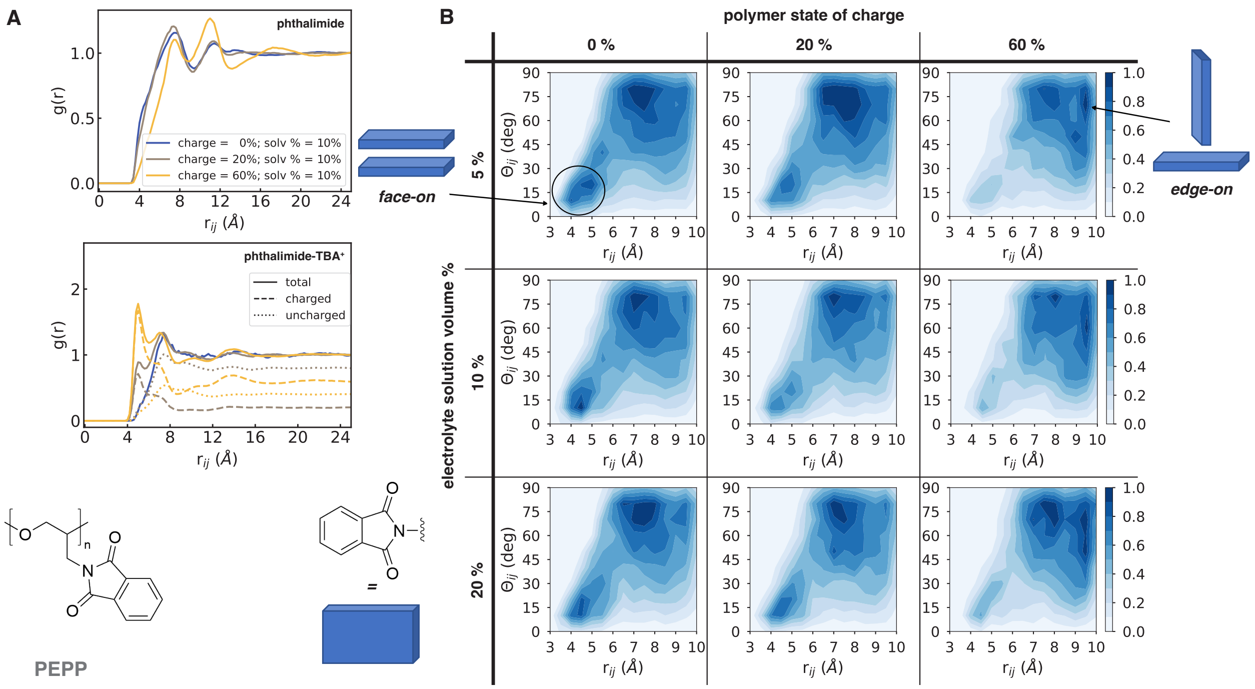

Figure S9: **Structural characterization of PEPP.** Same as Figure 3 in the main text but for PEPP.

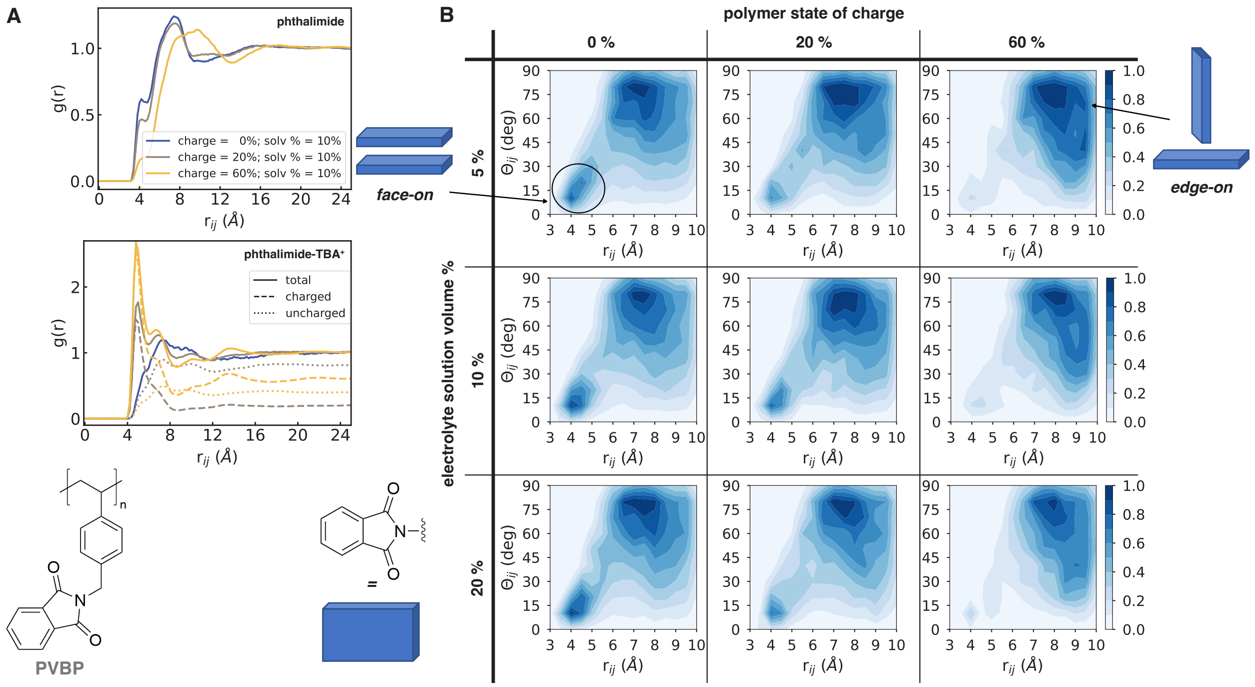

Figure S10: **Structural characterization of PVBP.** Same as Figure 3 in the main text but for PVBP.

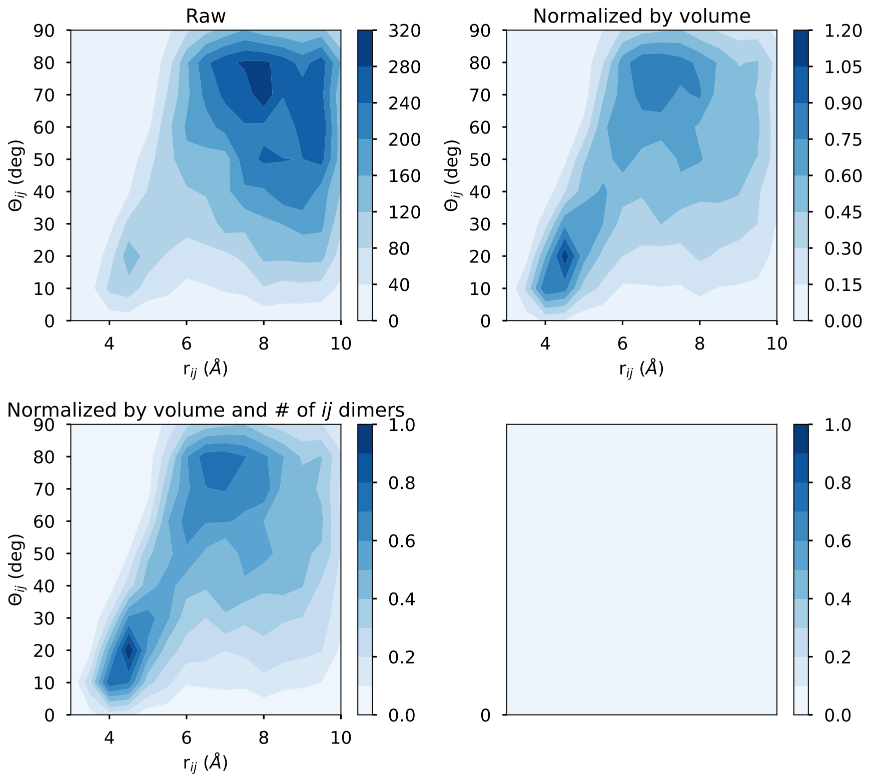

Figure S11: **Construction of the 2D configurational maps.** Binning of the phthalimide pairs according to their (r_{ij}, θ_{ij}) values results in the "raw" map (top-left). This map needs to be normalized to account for the increasing considered volume with increasing r_{ij} distances, and hence the higher chances of finding a neighbor with increasing r_{ij} distances. In 3D, this normalization is the volume of the spherical shell, $\rho 4\pi r^2 dr$; this normalization results in the map shown in the top-right. Finally, in order to compare 2D maps obtained from different systems, which can contain different amounts of phthalimide pairs, we normalize by the total number of ij pairs (bottom-right).

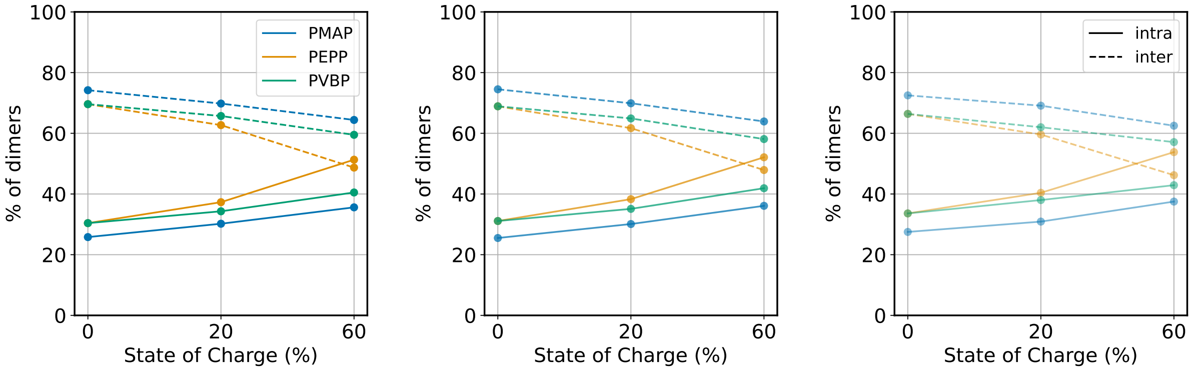

Figure S12: **Intramolecular vs. intermolecular pairs.** Solid lines indicate % of intramolecular pairs. Dashed lines indicate % of intermolecular pairs.

5 Ionic Diffusivity

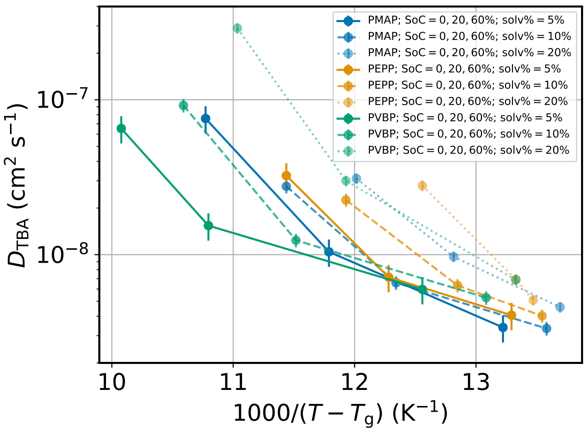

Figure S13: **TBA$^+$ ion diffusion coefficient, D_{TBA}, as a function of temperature, for the different states of charge and swelling %.** Data for each system are the ones at $T = 1.2 \cdot T_g$ reported in Figure 4A, but now plotted as a function of $1000/(T - T_g)$. D_{TBA} coefficients decrease as the distance from T_g decreases. Moreover, consistently with Figure 4A, the plot shows that D_{TBA} coefficients generally increase with increasing swelling %, while they decrease with increasing state of charge. Note that, in each 3-datapoint series, the left-hand side datapoint is SoC= 0%, the middle one SoC= 20%, and the right-hand side one is SoC= 60%.

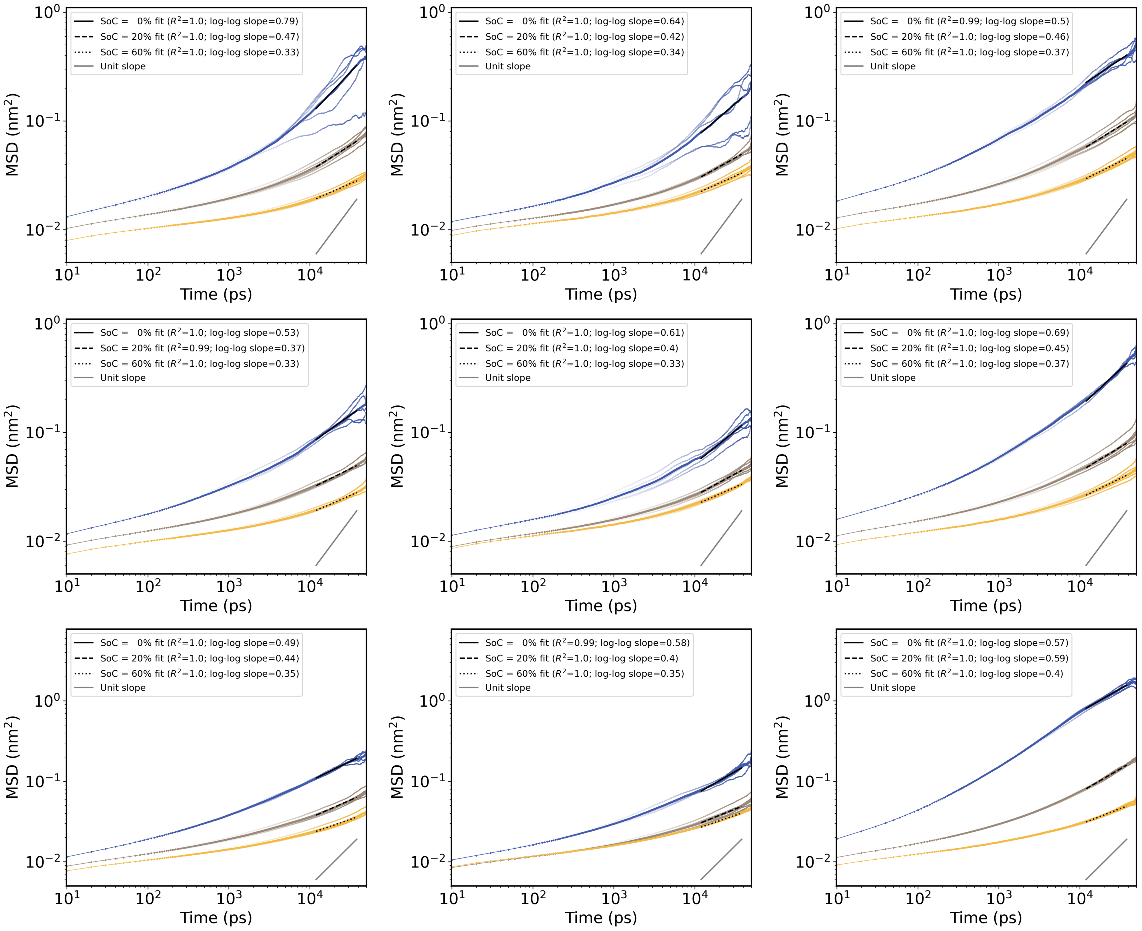

Figure S14: **Log-log plot of the mean-square displacement (MSD) of TBA$^+$.** (left) PMAP, (center) PEPP, (right) PVBP; (top) 5%, (middle) 10%, (bottom) 20% swelling. $T = 1.2 \cdot T_\mathrm{g}$. Each plot reports three MSDs, one for each state of charge (0% in blue, 20% in gray, 60% in yellow). Each of the MSD curve is an average (thick line) of 5 50-ns MSD curves (thin lines) taken from a 100 ns trajectory at different starting times. The slopes of the linear fits of the MSD average curve at the longest simulated times are reported in the legend. A guideline for the unit slope (diffusive regime) is also shown in each plot. The slopes (scaling exponents) are on average around 0.7 to around 0.4, with the systems with 0% state of charge being on average the closest to the diffusive regime, and the systems with 60% state of charge being the farthest. The Einstein relation is used to extract the diffusion coefficients in the highlighted region (the extracted diffusion coefficients are reported in Figure 4 of the main text).

6 Electronic Couplings

6.1 Orbital overlaps *vs.* electronic couplings

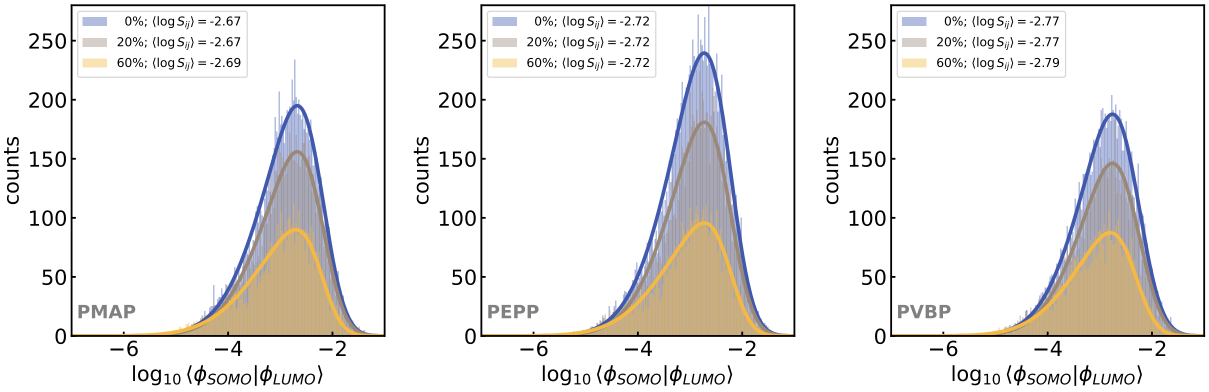

Figure S15: **Orbital overlap distributions as a function of state of charge for the different polymers.** Orbital overlap distributions corresponding to the electronic coupling distributions of Figure 5 of the main text. Mean of the overlap distributions, $\langle logS_{ij}\rangle$, obtained as the mode of the skew Gaussian distribution fits, are reported in the legends. Electrolyte solution volume % = 10%. $T = 300$ K.

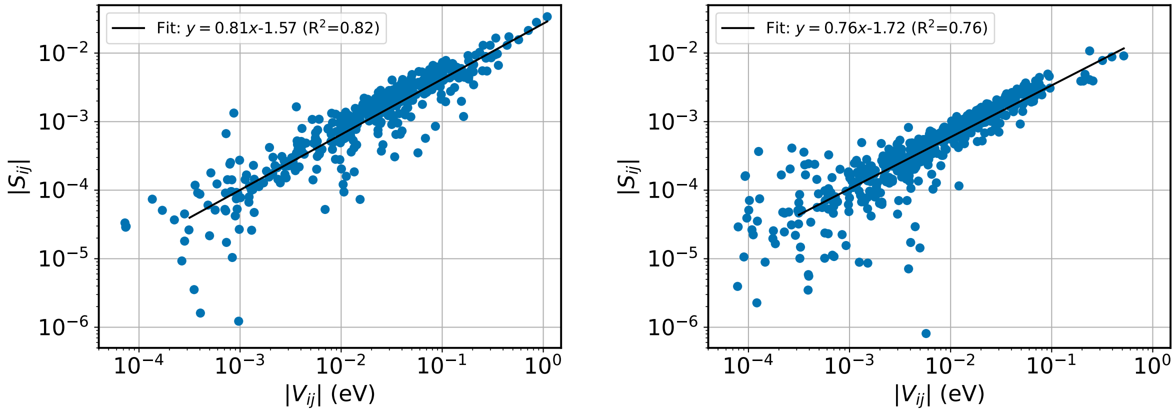

Figure S16: **Correlation between electronic couplings and orbital overlaps.** Correlation between DIPRO[S3] electronic couplings and orbital overalps. (left) N-methyl-phthalimide. (right) TEMPO. The linear fit parameters and R^2 score can be found in the legends.

6.2 Neural network surrogate model for orbital overlaps

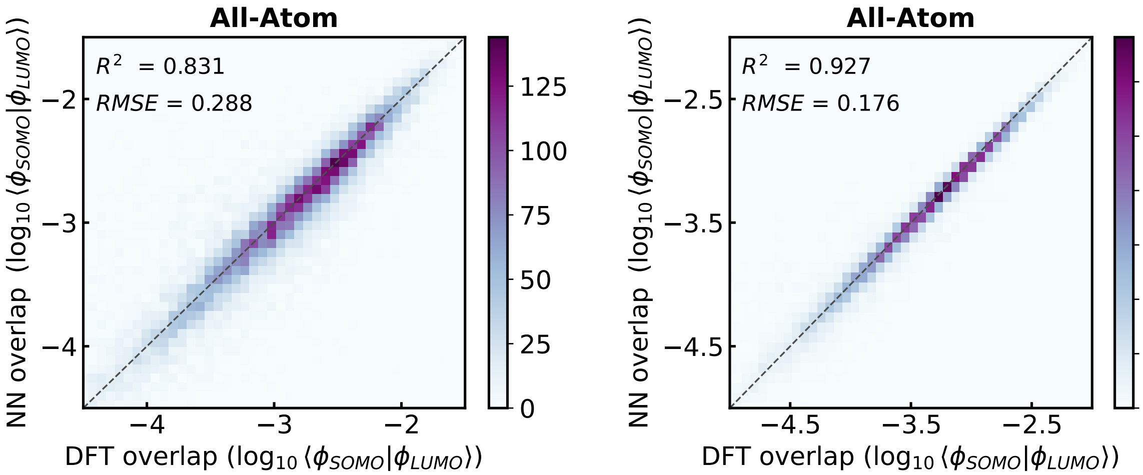

Figure S17: **Predictive performance of neural networks on the prediction of orbital overalps.** (left) N-methyl-phthalimide; (right) TEMPO. Two-dimensional histogram showing the accuracy achieved by the neural network (NN) surrogate model for DFT orbital overlap predictions. The R^2 score and RMSE are indicated on the plot and represent the results on the test set.

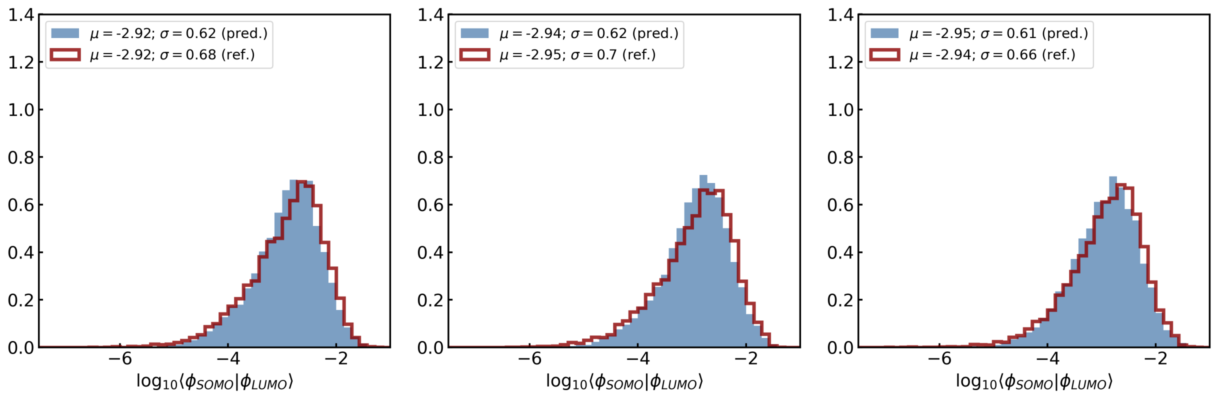

Figure S18: **Neural network predicted overlaps *vs.* reference values for three samples.** (left) PMAP, (center) PEPP, (right) PVBP. The distribution predicted by the neural network surrogate model is in blue, while the reference distribution of explicitly computed orbital overlaps is in red. Distributions are normalized.

7 Electronic Percolation

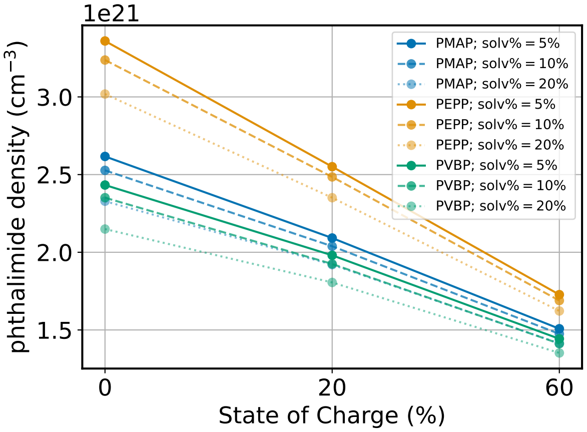

Figure S19: **Phthalimide density for the different systems.** Phthalimide number density as a function of polymer, state of charge, and swelling.

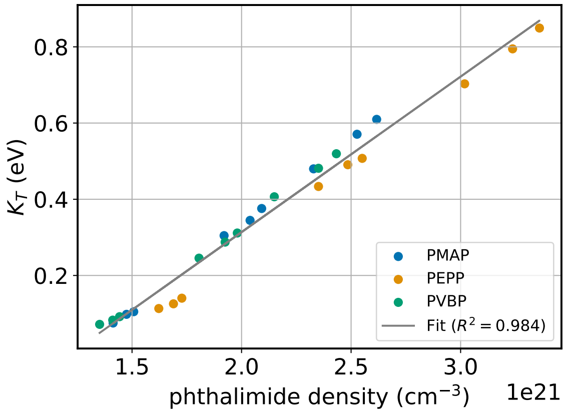

Figure S20: **Correlation between phthalimide density and K_T.** The linear fit is $K_\mathrm{T} = 4.08 \cdot 10^{-22} \rho_{NMePh} - 0.502$ ($R^2 = 0.984$).

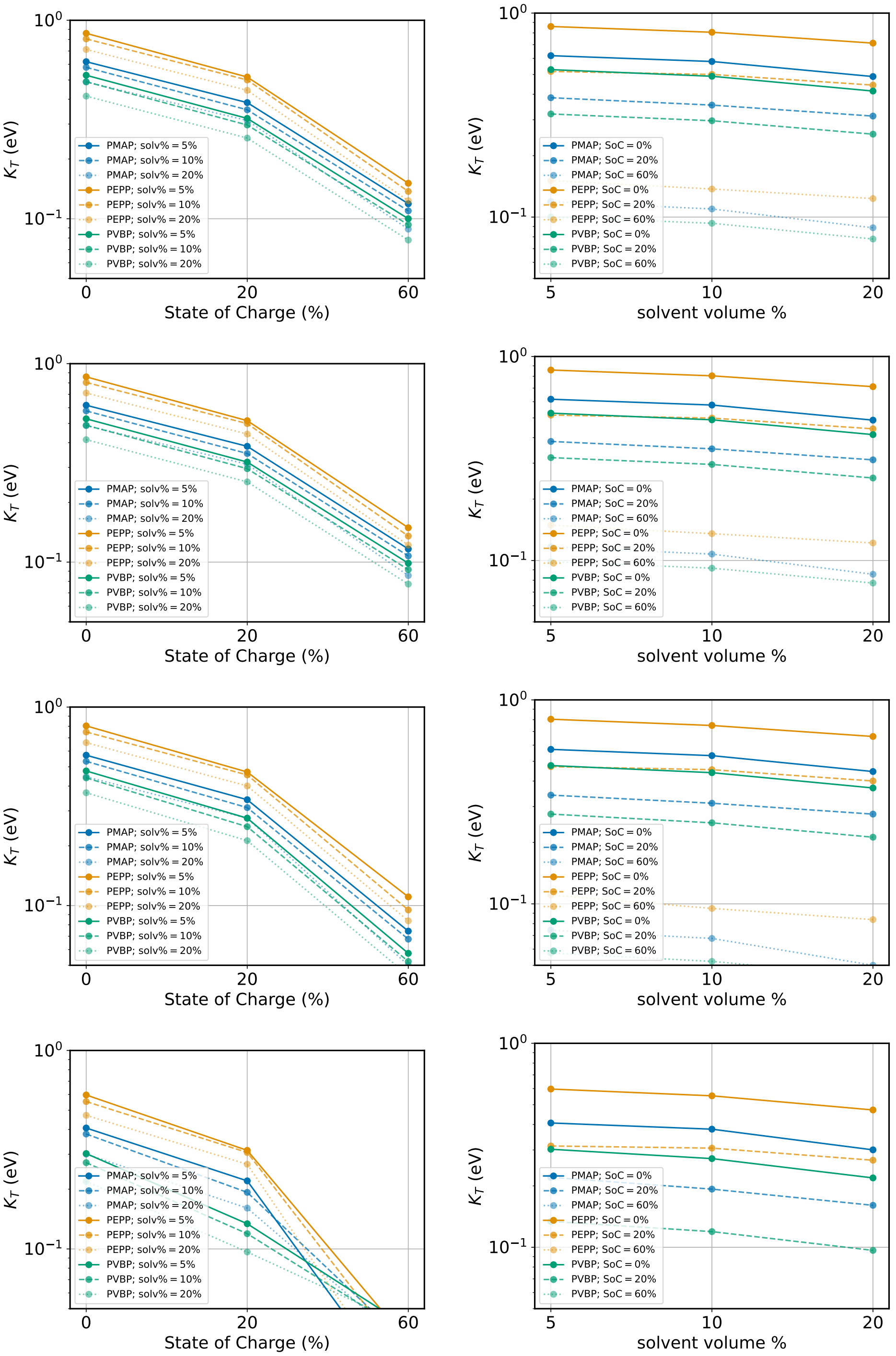

Figure S21: **Computed K_T as a function of electronic coupling threshold.** Comparison of computed K_T as a function of chosen threshold for the electronic couplings. From top to bottom thresholds are: 10, 50, 200, and 400 meV. Only when the threshold reaches the (rather high) value of 400 meV, some of the 60% results change qualitatively (bottom row).

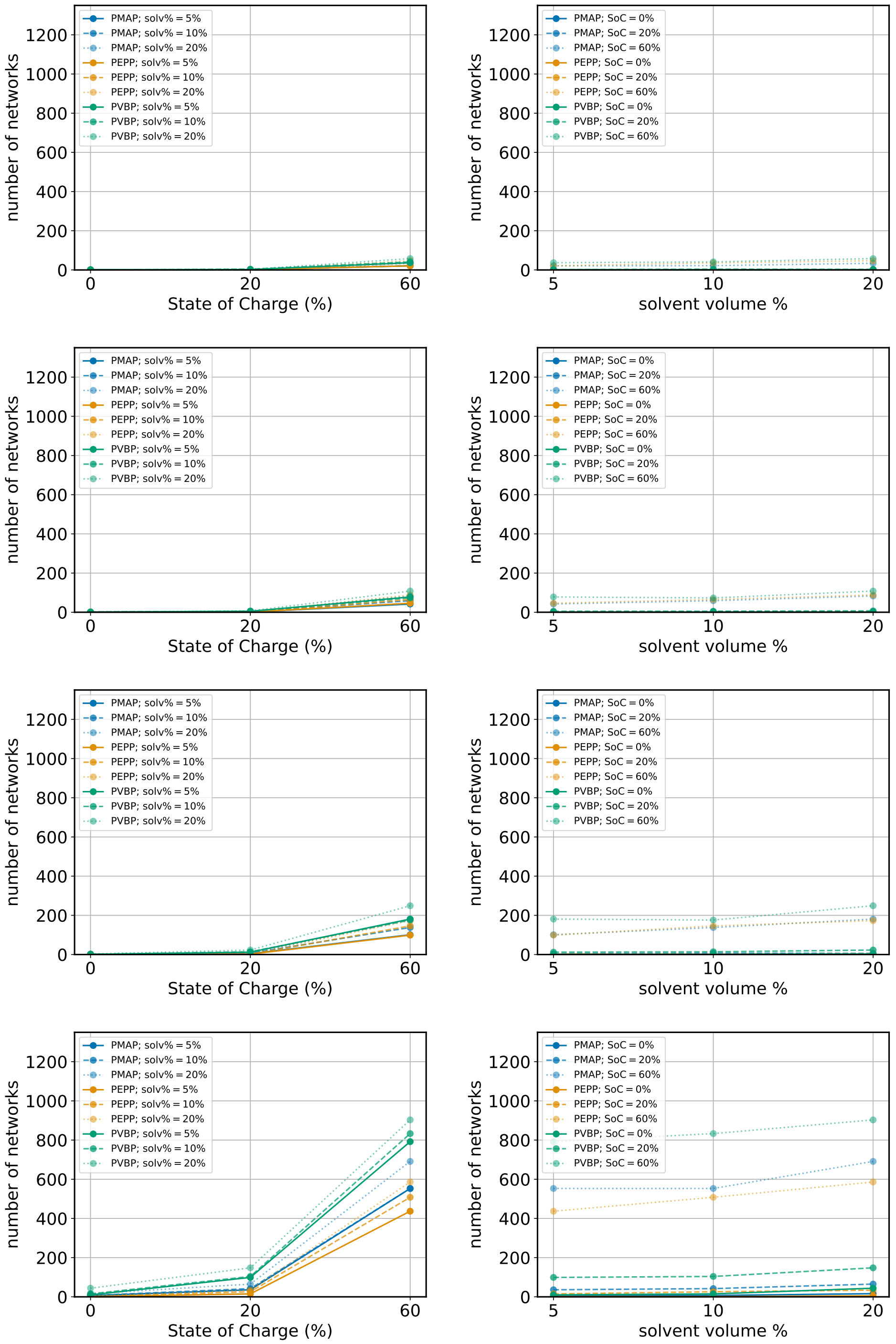

Figure S22: **Number of networks as a function of electronic coupling threshold.** Comparison of computed number of networks as a function of chosen threshold for the electronic couplings. From top to bottom thresholds are: 10, 100, 200, and 400 meV.

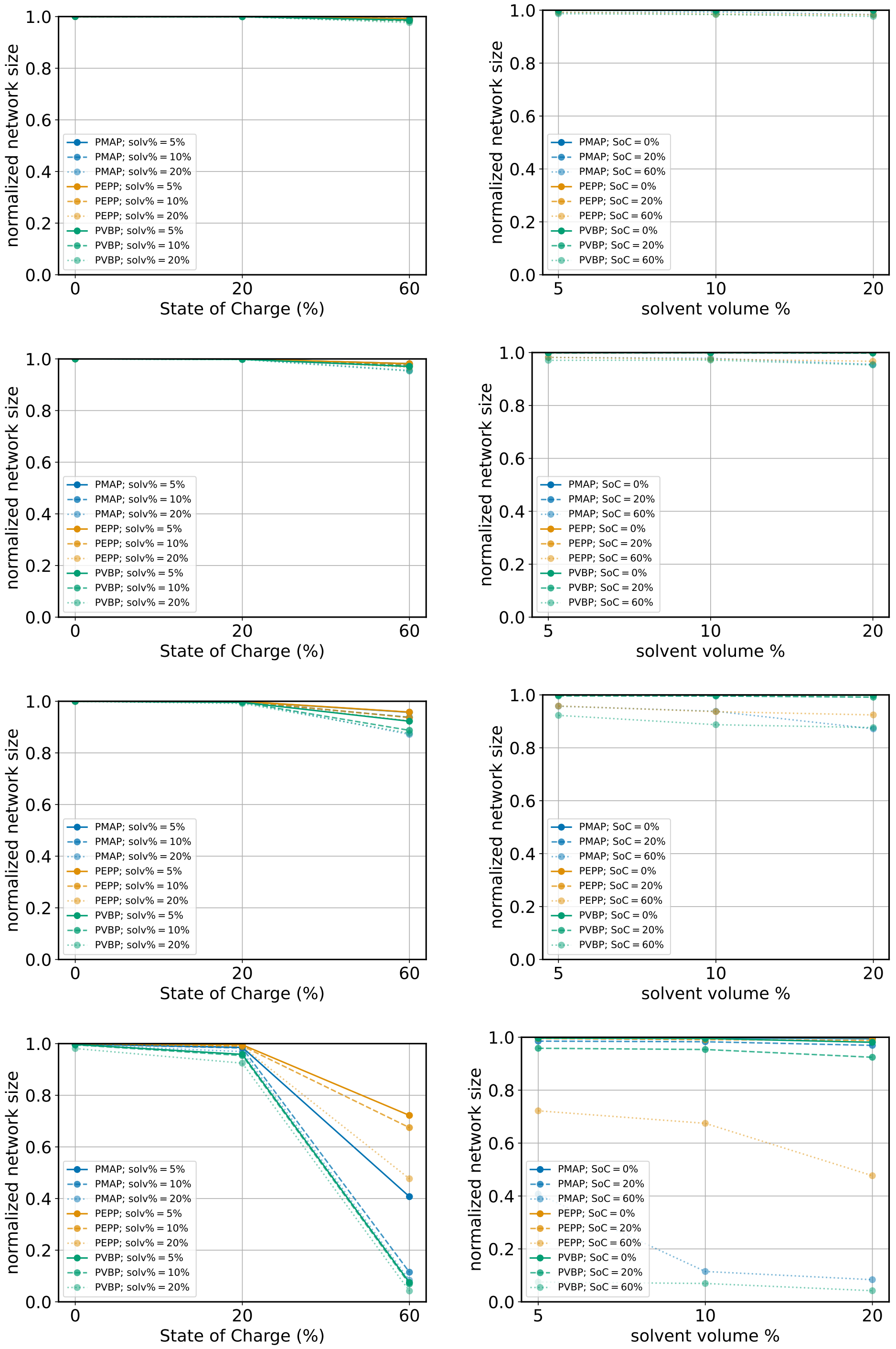

Figure S23: **Computed largest network size as a function of electronic coupling threshold.** Comparison of computed largest network size as a function of chosen threshold for the electronic couplings. From top to bottom thresholds are: 10, 100, 200, and 400 meV.

8 Connection to Experimental Observables

8.1 Phthalimide-phthalimide distance at electron transfer, δ (PMAP)

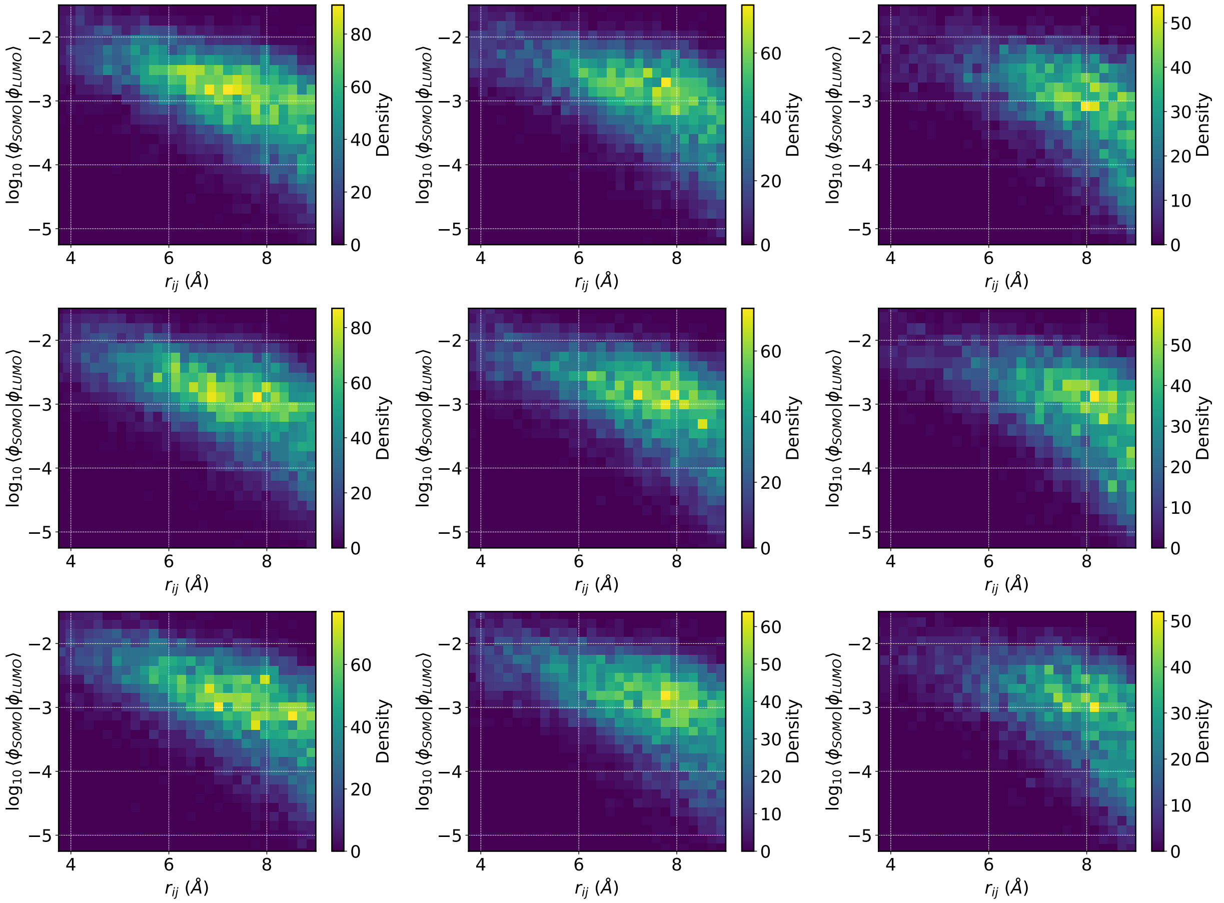

Figure S24: **Phthalimide interdistance r_{ij} *vs.* overlap heatmap, PMAP.** First row: swelling = 5%; second row: swelling = 10%; third row: swelling = 20%. First column: state of charge = 0%; second column: state of charge = 20%; third column: state of charge = 60%. $T = 300$ K. The center-to-center distance at electron transfer, δ, is computed as the orbital overlap-weighted mean distance: $\delta = \sum_n V_{ij} x_i / \sum_n V_{ij}$.

8.2 Phthalimide-phthalimide distance at electron transfer, δ (PEPP)

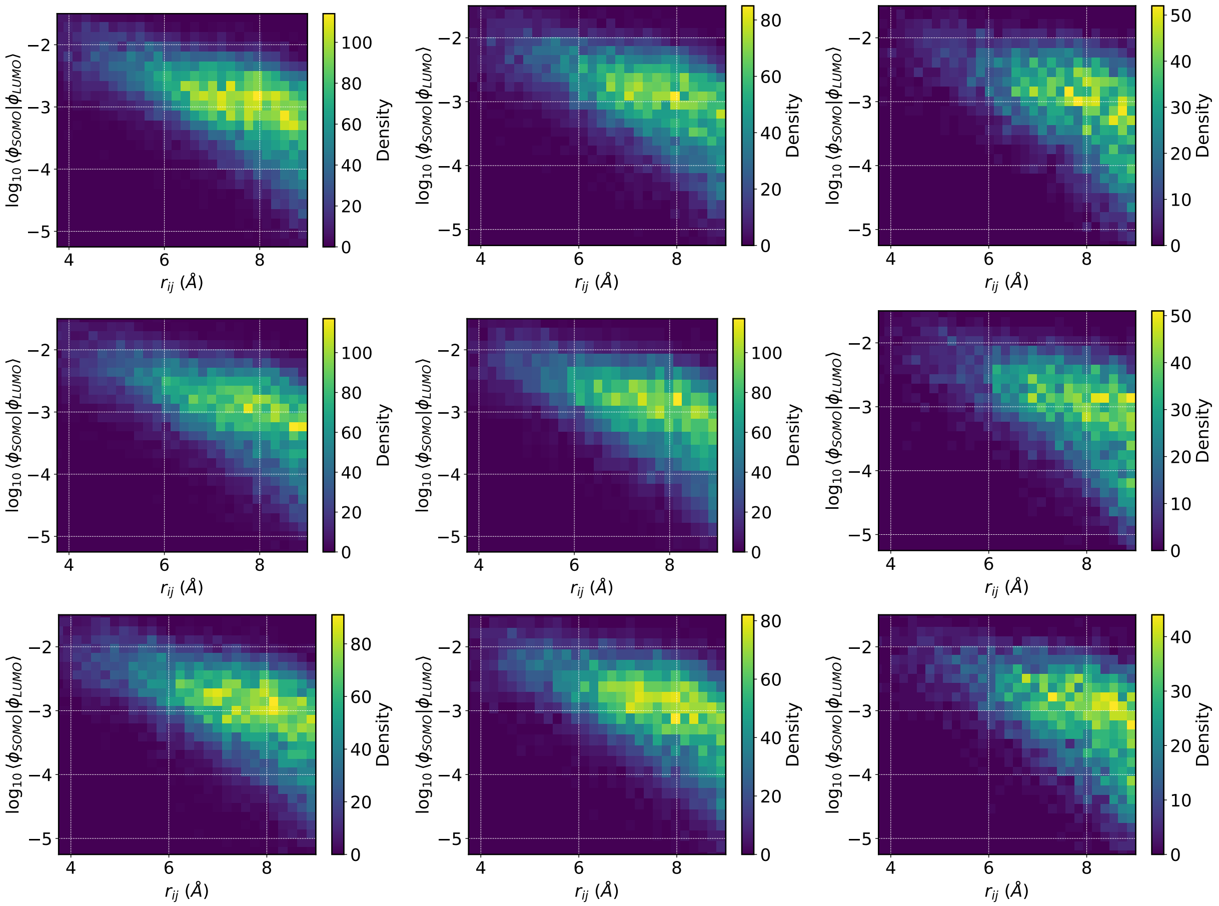

Figure S25: **Phthalimide interdistance r_{ij} *vs.* overlap heatmap, PEPP.** First row: swelling = 5%; second row: swelling = 10%; third row: swelling = 20%. First column: state of charge = 0%; second column: state of charge = 20%; third column: state of charge = 60%. T = 300 K. The center-to-center distance at electron transfer, δ, is computed as the orbital overlap-weighted mean distance: $\delta = \sum_n V_{ij} x_i / \sum_n V_{ij}$.

8.3 Phthalimide-phthalimide distance at electron transfer, δ (PVBP)

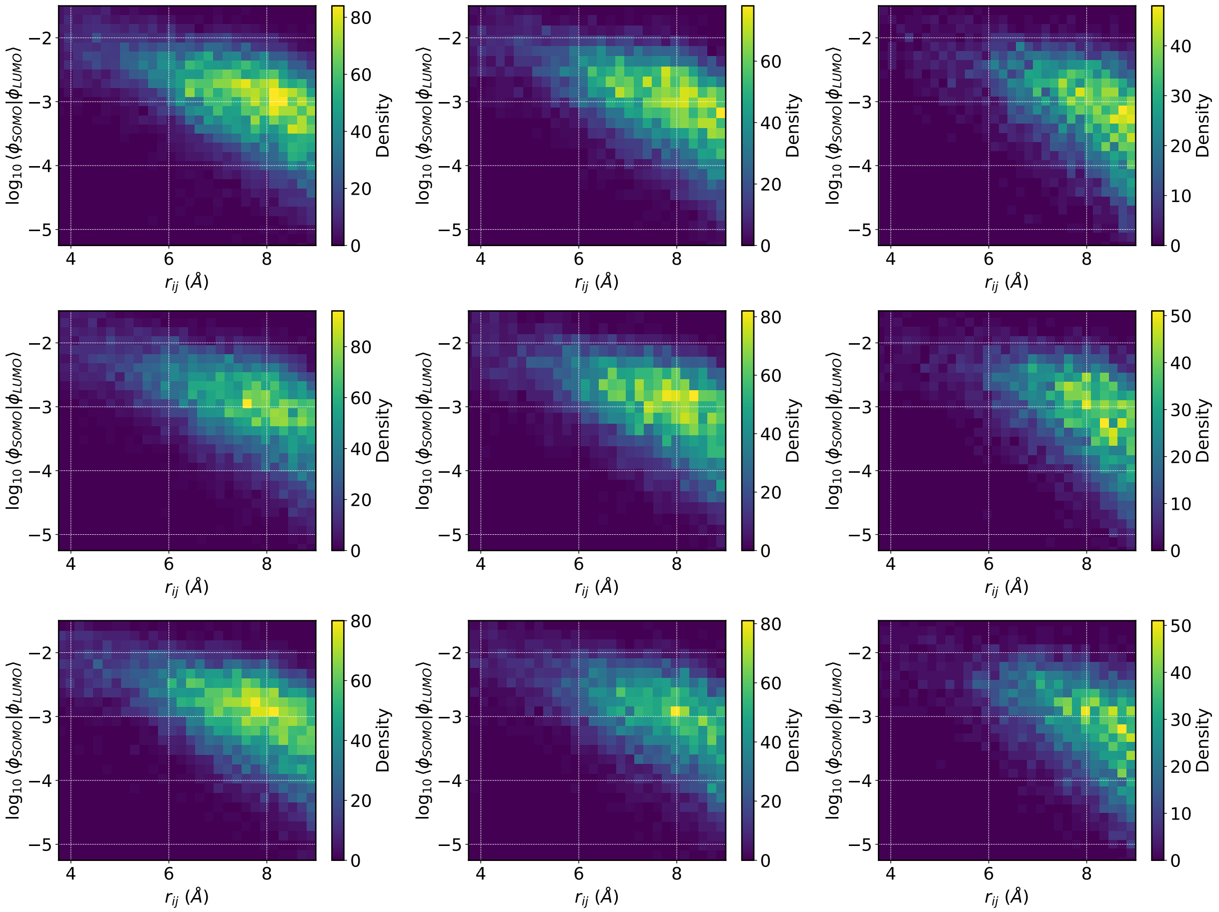

Figure S26: **Phthalimide interdistance r_{ij} *vs.* overlap heatmap, PVBP.** First row: swelling = 5%; second row: swelling = 10%; third row: swelling = 20%. First column: state of charge = 0%; second column: state of charge = 20%; third column: state of charge = 60%. T = 300 K. The center-to-center distance at electron transfer, δ, is computed as the orbital overlap-weighted mean distance: $\delta = \sum_n V_{ij}x_i / \sum_n V_{ij}$.

8.4 Reorganizatione energies

Table S2: **Reorganization energies.** Computed as described in the Methods section of the main text. All values are in eV.

	NMePh	PMAP	PEPP	PVBP	PTMA
λ_{in}	0.547	0.615	0.580	0.561	0.993
λ_{out}	0.419	0.419	0.419	0.419	0.507
λ	0.966	1.034	0.999	0.980	1.500

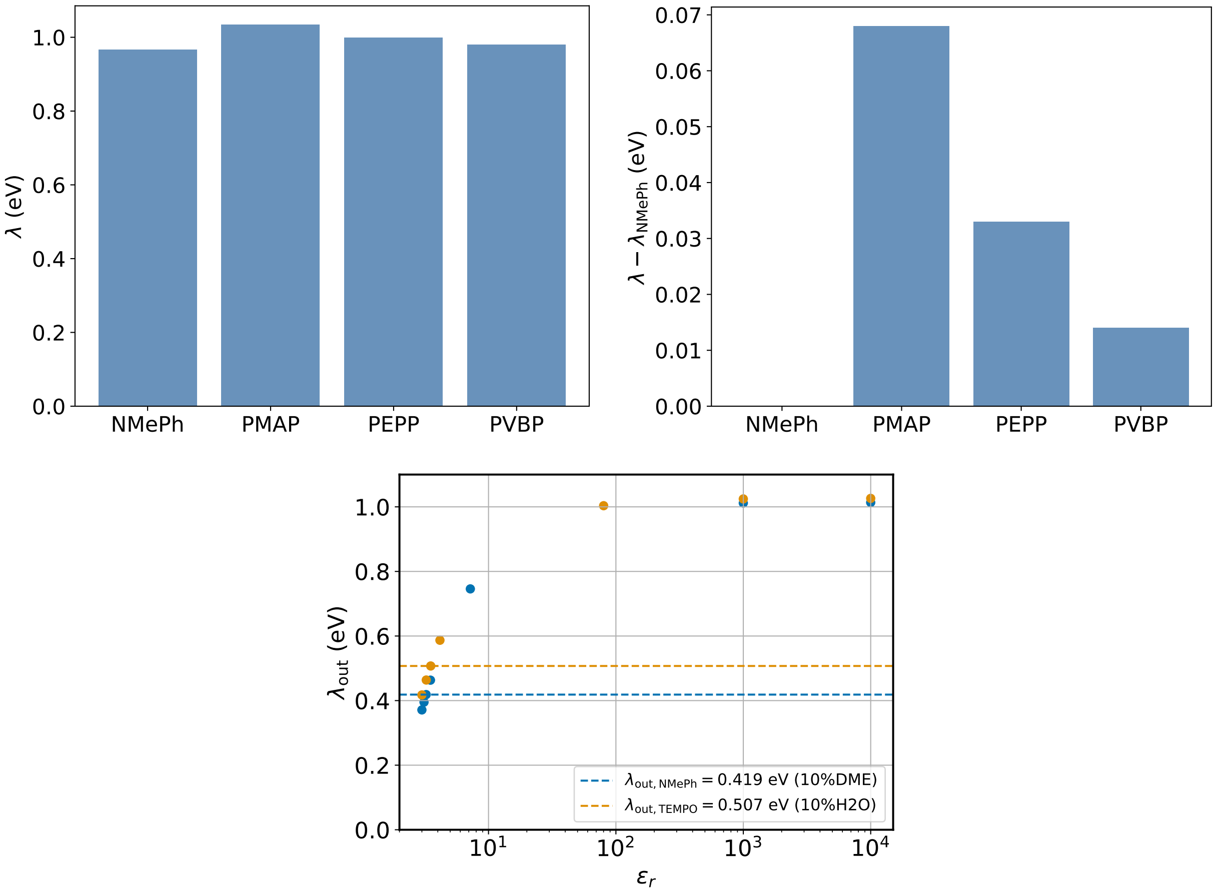

Figure S27: **Reorganization energies.** (top-left) Absolute values of λ for the different polymers and for N-methyl-phthalimide (NMePh). (top-right) Differences between the λ of NMePh, λ_{NMePh} and the λ values for the different polymers. Values computed at the ωB97X-D/def2-SV(P) level. (bottom) λ_{out} as a function of the relative permittivity of the environment, ε_r. The latter is computed using the following Debye model: $\frac{\varepsilon_r-1}{\varepsilon_r+2} = \Phi_1 \frac{\varepsilon_1-1}{\varepsilon_1+2} + \Phi_2 \frac{\varepsilon_2-1}{\varepsilon_2+2}$ where ε_1 and ε_2 are the relative permittivities of the polymer (assumed to be $\varepsilon_{\text{polymer}} = 3$ based on the experimental value for nitroxide radical-based polymers)[S4] and the solvent ($\varepsilon_{\text{DME}} = 7.2$ and $\varepsilon_{H_2O} = 80.1$),[S5] and Φ_1 and Φ_2 are the volume fractions of the polymer and solvent, respectively.

8.5 Additional results for the apparent diffusion coefficient, D_{app}

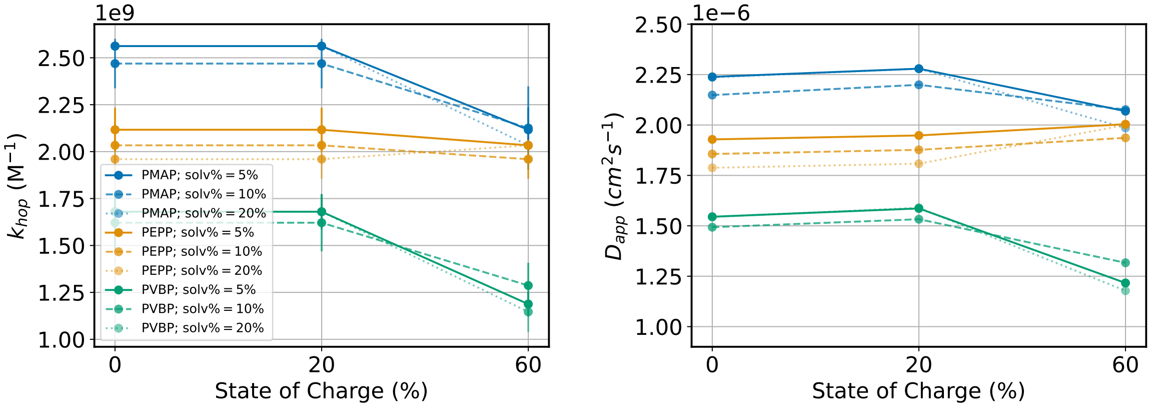

Figure S28: **Predicted electron transport performance of systems investigated ($\lambda = \lambda_{\text{NMePh}}$).** Same as Figure 7 but with $\lambda = \lambda_{\text{NMePh}}$ for all polymers.

8.6 Apparent diffusion coefficient (D_{app}) of PTMA

Table S3: **Experimental D_{app} for PTMA systems.** [NO] = concentration of nitroxide radicals; TEA = tetraethylammonium; BF_4 = tetrafluoroborate; ClO_4 = perchlorate; EC = ethylene carbonate; DEC = diethyl carbonate; ACN = acetonitrile.

	[NO] (M)	[salt] (M)	D_{app} (cm^2 s^{-1})	$k_{ex,app}$ (M^{-1} s^{-1})	Ref.
PTMA:H_2O:$TEABF_4$	3.7	0.5	$4.0 \cdot 10^{-10}$	$1.1 \cdot 10^5$	S6
PTMA:EC/DEC(3/7 v/v):$LiClO_4$	—[a]	0.1	$3.1 \cdot 10^{-11}$	—	S7
PTMA:ACN:$TBAClO_4$	3.12	0.5	—[a]	$1.8 \cdot 10^4$	S8

[a] Value not reported.

Table S4: **Predicted D_{app}, k_{hop}, values for the PTMA:H_2O:$TEABF_4$ solid-state-system.** [NO] = concentration of nitroxide radicals.

	swelling %	[NO] (M)	[$TEABF_4$] (M)	D_{app} (cm^2 s^{-1})	k_{hop} (s^{-1})	V_{ij} (meV)	δ Å
PTMA:H_2O:$TEABF_4$	5	4.0	0.5	$4.8 \cdot 10^{-10}$	$4.4 \cdot 10^5$	8.4	8.05
PTMA:H_2O:$TEABF_4$	10	3.8	0.5	$4.8 \cdot 10^{-10}$	$4.4 \cdot 10^5$	8.4	8.06
PTMA:H_2O:$TEABF_4$	20	3.4	0.5	$4.8 \cdot 10^{-10}$	$4.4 \cdot 10^5$	8.4	8.10

[a] Value not reported.

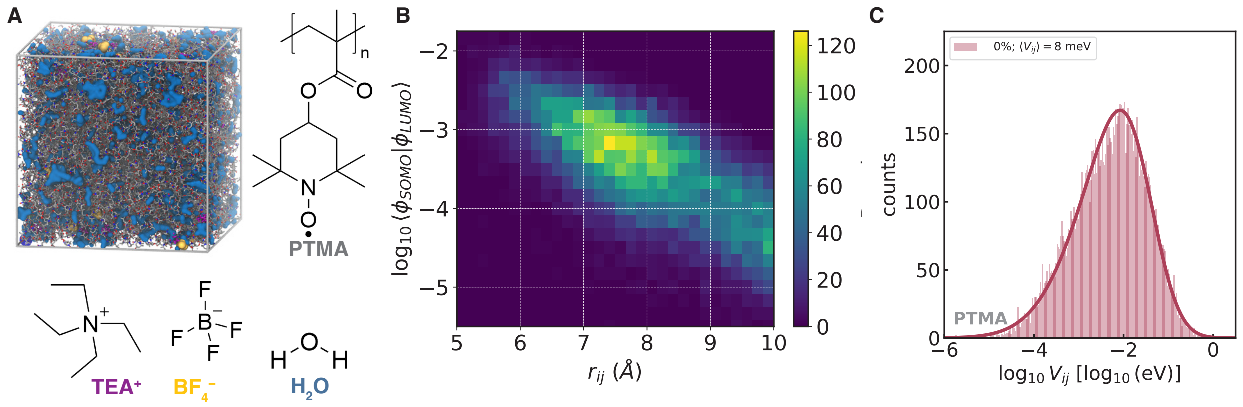

Figure S29: D_{app} **for PTMA.** (**A**) Rendering of the PTMA:H_2O:$TEABF_4$ system swollen at 10% swelling. (**B**) TEMPO interdistance r_{ij} *vs.* overlap heatmap for PTMA. The center-to-center distance at electron transfer, δ, is computed as the orbital overlap-weighted mean distance: $\delta = \sum_n V_{ij}x_i / \sum_n V_{ij}$; shown is the map for PTMA:H_2O:$TEABF_4$ at 10% swelling; see Table S4 for the obtained δ values. (**C**) Electronic coupling distribution for the PTMA:H_2O:$TEABF_4$ at 10% swelling. The mean electronic couplings, $\langle V_{ij} \rangle$, obtained as the mode of the skew Gaussian distribution fit, is reported in the legend. $T = 300$ K.

References

(S1) Alessandri, R.; de Pablo, J. J. Prediction of Electronic Properties of Radical-Containing Polymers at Coarse-Grained Resolutions. *Macromolecules* **2023**, *56*, 3574–3584.

(S2) Grünewald, F.; Alessandri, R.; Kroon, P. C.; Monticelli, L.; Souza, P. C. T.; Marrink, S. J. Polyply; a python suite for facilitating simulations of macromolecules and nanomaterials. *Nat. Commun.* **2022**, *13*, 68.

(S3) Baumeier, B.; Kirkpatrick, J.; Andrienko, D. Density-functional based determination of intermolecular charge transfer properties for large-scale morphologies. *Phys. Chem. Chem. Phys.* **2010**, *12*, 11103–11113.

(S4) Baradwaj, A. G.; Rostro, L.; Alam, M. A.; Boudouris, B. W. Quantification of the solid-state charge mobility in a model radical polymer. *Appl. Phys. Lett.* **2014**, *104*, 213306.

(S5) Haynes, W. M. *CRC handbook of chemistry and physics*; CRC press, 2014.

(S6) Ma, T.; Easley, A. D.; Wang, S.; Flouda, P.; Lutkenhaus, J. L. Mixed electron-ion-water transfer in macromolecular radicals for metal-free aqueous batteries. *Cell Rep. Phys. Sci.* **2021**, *2*, 100414.

(S7) Tokue, H.; Murata, T.; Agatsuma, H.; Nishide, H.; Oyaizu, K. Charge–Discharge with Rocking-Chair-Type Li+ Migration Characteristics in a Zwitterionic Radical Copolymer Composed of TEMPO and Trifluoromethanesulfonylimide with Carbonate Electrolytes for a High-Rate Li-Ion Battery. *Macromolecules* **2017**, *50*, 1950–1958.

(S8) Sato, K.; Ichinoi, R.; Mizukami, R.; Serikawa, T.; Sasaki, Y.; Lutkenhaus, J.; Nishide, H.; Oyaizu, K. Diffusion-Cooperative Model for Charge Transport by Redox-Active Nonconjugated Polymers. *J. Am. Chem. Soc.* **2018**, *140*, 1049–1056.
